# Cross-communication between G_i_ and G_s_ in a G-protein-coupled receptor heterotetramer guided by a receptor C-terminal domain

**DOI:** 10.1186/s12915-018-0491-x

**Published:** 2018-02-28

**Authors:** Gemma Navarro, Arnau Cordomí, Marc Brugarolas, Estefanía Moreno, David Aguinaga, Laura Pérez-Benito, Sergi Ferre, Antoni Cortés, Vicent Casadó, Josefa Mallol, Enric I. Canela, Carme Lluís, Leonardo Pardo, Peter J. McCormick, Rafael Franco

**Affiliations:** 1 Centro de Investigación Biomédica en Red sobre Enfermedades Neurodegenerativas, University of Barcelona, 08028 Barcelona, Spain; 2 Institute of Biomedicine of the University of Barcelona (IBUB), University of Barcelona, 08028 Barcelona, Spain; 3 Department of Biochemistry and Molecular Biomedicine, Faculty of Biology, University of Barcelona, 08028 Barcelona, Spain; 4 Laboratori de Medicina Computacional, Unitat de Bioestadística, Facultat de Medicina, Universitat Autònoma de Barcelona, 08193 Bellaterra, Spain; 5 Integrative Neurobiology Section, National Institute on Drug Abuse, National Institutes of Health, Baltimore, MD 21224 USA; 6 School of Veterinary Medicine, University of Surrey, Guildford, Surrey, GU2 7AL UK

**Keywords:** C-terminal domain, GPCR, Heterotetramer, BRET, Molecular modeling

## Abstract

**Background:**

G-protein-coupled receptor (GPCR) heteromeric complexes have distinct properties from homomeric GPCRs, giving rise to new receptor functionalities. Adenosine receptors (A_1_R or A_2A_R) can form A_1_R-A_2A_R heteromers (A_1_-A_2A_Het), and their activation leads to canonical G-protein-dependent (adenylate cyclase mediated) and -independent (β-arrestin mediated) signaling. Adenosine has different affinities for A_1_R and A_2A_R, allowing the heteromeric receptor to detect its concentration by integrating the downstream G_i_- and G_s_-dependent signals. cAMP accumulation and β-arrestin recruitment assays have shown that, within the complex, activation of A_2A_R impedes signaling via A_1_R.

**Results:**

We examined the mechanism by which A_1_-A_2A_Het integrates G_i_- and G_s_-dependent signals. A_1_R blockade by A_2A_R in the A_1_-A_2A_Het is not observed in the absence of A_2A_R activation by agonists, in the absence of the C-terminal domain of A_2A_R, or in the presence of synthetic peptides that disrupt the heteromer interface of A_1_-A_2A_Het, indicating that signaling mediated by A_1_R and A_2A_R is controlled by both G_i_ and G_s_ proteins.

**Conclusions:**

We identified a new mechanism of signal transduction that implies a cross-communication between G_i_ and G_s_ proteins guided by the C-terminal tail of the A_2A_R. This mechanism provides the molecular basis for the operation of the A_1_-A_2A_Het as an adenosine concentration-sensing device that modulates the signals originating at both A_1_R and A_2A_R.

**Electronic supplementary material:**

The online version of this article (10.1186/s12915-018-0491-x) contains supplementary material, which is available to authorized users.

## Background

Adenosine is a purine nucleoside whose relevance in the central nervous system is mainly due to its role in regulating neurotransmitter release [1]. The effects of adenosine are mediated by specific G-protein-coupled receptors (GPCRs) that are coupled to either G_s_ or G_i_ heterotrimeric G_αβγ_ proteins. The endogenous adenosine acts on four receptor subtypes – A_1_R, A_2A_R, A_2B_R, and A_3_R. Convergent and compelling evidence shows that GPCRs may form complexes constituted by a number of equal (homo) or different (hetero) receptor protomers [2]. As agreed in the field, a GPCR heteromer displays characteristics that are different from those of the constituting protomers, thus giving rise to novel functional entities [3]. Adenosine receptors have been used as a paradigm in the study of receptor homo- and heteromerization. For instance, A_1_R, which is G_i_ coupled, and A_2A_R, which is G_s_ coupled, form a functional heteromer [4].

The A_1_R-A_2A_R heteromer (A_1_-A_2A_Het) is found presynaptically in, inter alia, cortical glutamatergic terminals innervating the striatum and functions as a switch that differentially senses high and low concentrations of adenosine in the inter-synaptic space. Since adenosine has higher affinity for A_1_R than for A_2A_R, low concentrations predominantly activate A_1_R, engaging a G_i_-mediated signaling, whereas higher adenosine concentrations also activate A_2A_R, engaging a G_s_-mediated signaling [4]. The physiological role of such a concentration-sensing device is remarkable as it allows adenosine to fine-tune modulate the release of neurotransmitters from presynaptic terminals. However, the mechanism by which A_1_-A_2A_Het integrates both G_i_- and G_s_-dependent signals is not yet understood. We have recently shown, using a combination of single-particle tracking experiments, bioluminescence resonance energy transfer (BRET) assays, and computer modeling, that the (minimal) functional A_1_-A_2A_Het/G protein unit is composed by a compact rhombus-shaped heterotetramer (with A_1_R and A_2A_R homodimers) bound to two different interacting heterotrimeric G proteins (G_s_ and G_i_) [5]. In the present study, we aim to understand the molecular intricacies underlying the signaling mediated by A_1_-A_2A_Het, in which (1) both receptors constituting the heteromer are activated by the same endogenous agonist and (2) is coupled to two different G proteins with opposite effects, i.e., one mediating the inhibition of the adenylate cyclase (G_i_) and another mediating the activation of the enzyme (G_s_). Our data identifies a new mechanism of signal transduction and provides the molecular basis to understand the unique properties of this heteromer, in which the C-terminal tail of the A_2A_R influences the G_i_-mediated signaling of the partner A_1_R receptor.

## Results

### Homodimerization of A_1_R and A_2A_R occurs through the transmembrane (TM) 4/5 interface and heterodimerization via the TM5/6 interface in the A_1_-A_2A_Het

**Fig. 1:**
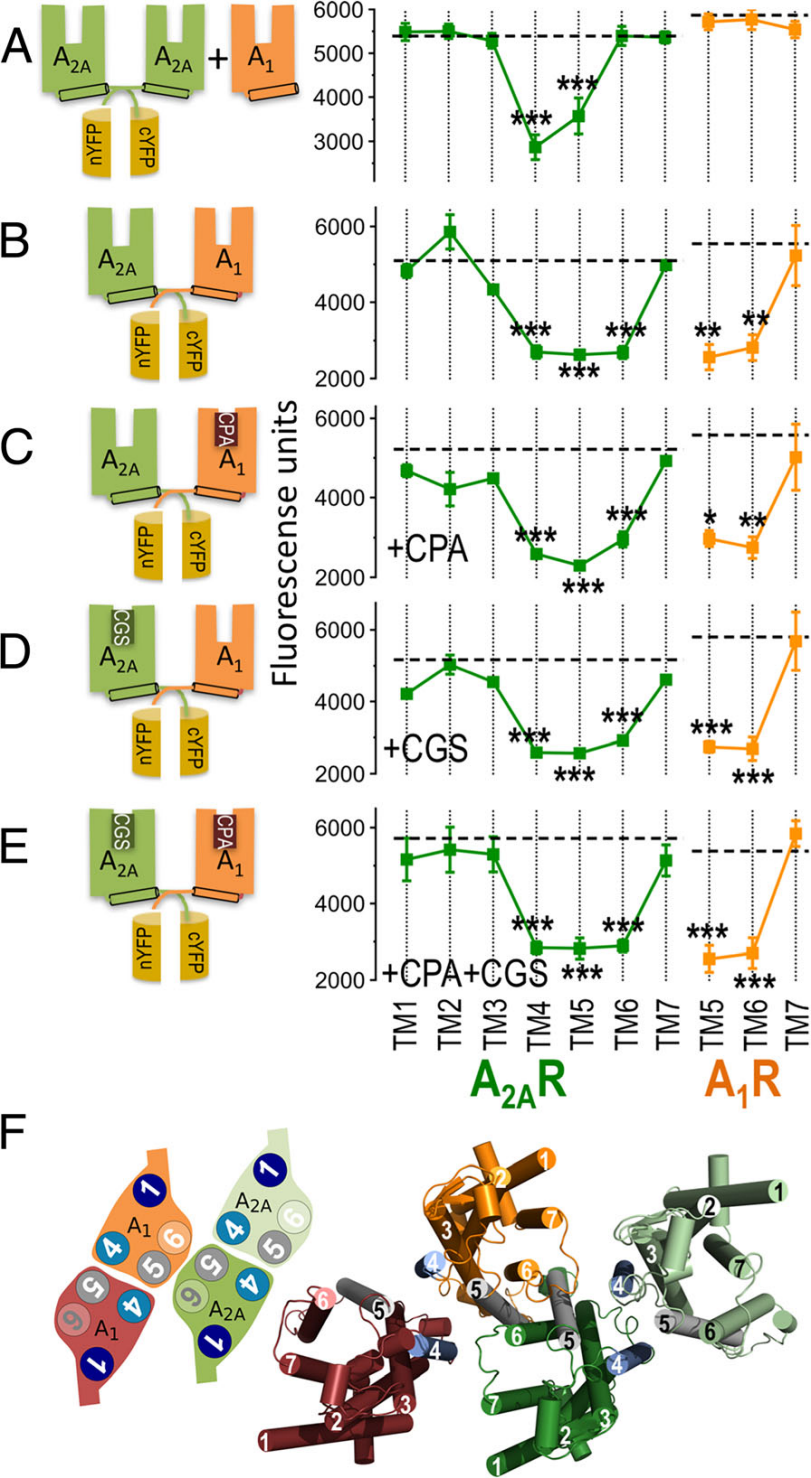
Effect of interference peptides on the A_1_-A_2A_Het structure determined by bimolecular fluorescence complementation (BiFC) assays. **a**–**e** BiFC assays were performed in HEK-293 T cells transfected with cDNAs (1 μg) for A_2A_R-nYFP, A_2A_R-cYFP, and non-fused A_1_R (**a**) or A_2A_R-cYFP and A_1_R-nYFP (**b**–**e**). Cells were pre-treated for 4 h with medium (control, broken lines) or with 4 μM of A_2A_R TM synthetic peptides (TM1 to TM7, green squares) or A_1_R synthetic peptides (TM5 to TM7, orange squares). Subsequently, they were left untreated (**a**, **b**) or activated for 10 min with the A_1_R agonist CPA (**c**, 100 nM), the A_2A_R agonist CGS-21680 (**d**, 100 nM), or both (**e**). Fluorescence was read at 530 nm. Mean ± SEM (13 experiments/treatment). One-way ANOVA followed by a Dunnett’s multiple comparison test showed a significant fluorescence decrease over control values (*P < 0.05, **P < 0.01, ***P < 0.001). In each panel, there is a schematic representation of the BiFC pairs and conditions. (**f**) Schematic slice (left) and cartoon (right) representations of the A_1_-A_2A_Het built using the predicted experimental interfaces

Our recently published BRET-aided computational model of the A_1_-A_2A_Het predicted the TM interfaces involved in homo- (TM4/5) and heterodimerization (TM5/6) [5]. To further confirm this arrangement, we used synthetic peptides with the sequence of TM domains of the A_2A_R (abbreviated TM1 to TM7) and the A_1_R (abbreviated TM5 to TM7), fused to the cell-penetrating HIV transactivator of transcription (TAT) peptide [6], to alter inter-protomer interactions in the A_1_-A_2A_Het. These peptides were first tested in bimolecular fluorescence complementation (BiFC) assays in HEK-293 T cells expressing receptors fused to two complementary halves of YFP (cYFP and nYFP) (see Methods). We detected fluorescence in HEK-293 T cells transfected with cDNAs for A_2A_R-nYFP, A_2A_R-cYFP, and non-fused A_1_R (broken lines in Fig. 1a), indicating the formation of the A_2A_R-A_2A_R homodimer. Notably, in the presence of interference peptides, we observed a fluorescence decrease only with TM4 and TM5 of A_2A_R (Fig. 1a), but not with A_1_R TM peptides (Fig. 1a) or with peptides derived from the orexin receptor (Additional file 1: Figure S1A) used as negative controls. Further negative controls show that A_2A_R peptides do not alter fluorescence in HEK-293 T cells expressing A_1_R-nYFP and A_1_R-cYFP (Additional file 1: Figure S1B). These results therefore confirmed the TM4/5 interface for A_2A_R homodimerization in the heteromer. Similarly, we detected fluorescence in cells expressing A_1_R-nYFP and A_2A_R-cYFP (broken lines in Fig. 1b), indicating formation of the A_1_-A_2A_Het. This fluorescence was only reduced in the presence of TM4, TM5, and TM6 peptides of A_2A_R (Fig. 1b). The involvement of TM5/6 in the heteromer interface was also confirmed by the fact that TM5 and TM6, but not TM7, of A_1_R reduced fluorescence in cells expressing A_1_R-nYFP and A_2A_R-cYFP (Fig. 1b). These results reinforce our previously proposed compact rhombus-shaped arrangement of protomers in which heteromerization of A_1_-A_2A_Het occurs via the TM5/6 interface (Fig. 1f). The fluorescence decrease induced by TM4 A_2A_R peptide indicates that the correct homomerization is a requisite for A_1_-A_2A_Het formation and/or that the TM4 peptide interferes with interactions of the TM4 of the external protomer of the A_2A_R homodimer with the internal protomer of the A_1_R homodimer (Fig. 1f) [5]. Next, we evaluated whether receptor activation, by the A_1_R-selective agonist N^6^-cyclopentyladenosine (CPA), the A_2A_R-selective agonist 4-[2-[[6-Amino-9-(N-ethyl-β-D-ribofuranuronamidosyl)-9H-purin-2-yl]amino]ethyl] benzenepropanoic acid (CGS-21680), or both, modify the heteromer TM interface. As clearly shown in Figs. 1c–e, none of the agonists, used either individually (Figs. 1c, d) or in combination (Fig. 1e), modified the effect of the TM peptides relative to the ligand-free experiments. Therefore, no rearrangements of the TM interface in the A_1_-A_2A_Het occurred upon receptor activation.

**Fig. 2:**
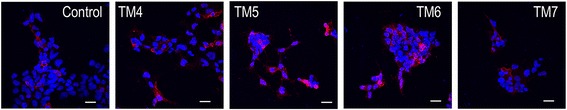
Effect of interference peptides on the A_1_-A_2A_Het structure determined by proximity ligation assay (PLA) confocal microscopy images (superimposed sections) in which A_1_-A_2A_Hets appear as red spots. HEK-293 T cells expressing A_1_R and A_2A_R were treated for 4 h with medium (control) or 4 μM of indicated TM peptides of A_2A_R; cell nuclei were stained with DAPI (blue); scale bars: 10 μm

Next, we investigated whether interference TM peptides, which are able to alter the quaternary structure of the A_1_-A_2A_Het as demonstrated by BiFC experiments, are also able to disrupt the heteromer. To do this, proximity ligation assays (PLA) were performed in HEK-293 T cells expressing A_1_R and A_2A_R. The PLA assay is a powerful technique to detect protein-protein interactions by assessing proximity between GPCR protomers with high resolution (< 40 nm). A_1_-A_2A_Het was observed as red punctate staining (Fig. 2), whereas pretreatment of cells with TM4, TM5, TM6, and TM7 of A_2A_R did not decrease PLA staining (Fig. 2), indicating that interference peptides can alter the quaternary structure of the heteromer but cannot disrupt heteromerization.

### The complex formed by G_s_, G_i_, and the A_1_-A_2A_Het as a signal transduction unit

In order to test the ability of G_s_ and G_i_ proteins to interact with the A_1_-A_2A_Het, we used BRET assays [7]. Cells were transfected with cDNAs of A_1_R-nYFP and A_2A_R-cYFP, which only upon complementation can act as a BRET acceptor (YFP), and *Renilla* luciferase (Rluc) as a BRET donor fused to either G_i_ (G_i_-Rluc) or G_s_ (G_s_-Rluc). We observed significant energy transfer (Additional file 1: Figure S1C), indicating that G_i_ and G_s_ are bound to their respective receptors in the A_1_-A_2A_Het.

**Fig. 3:**
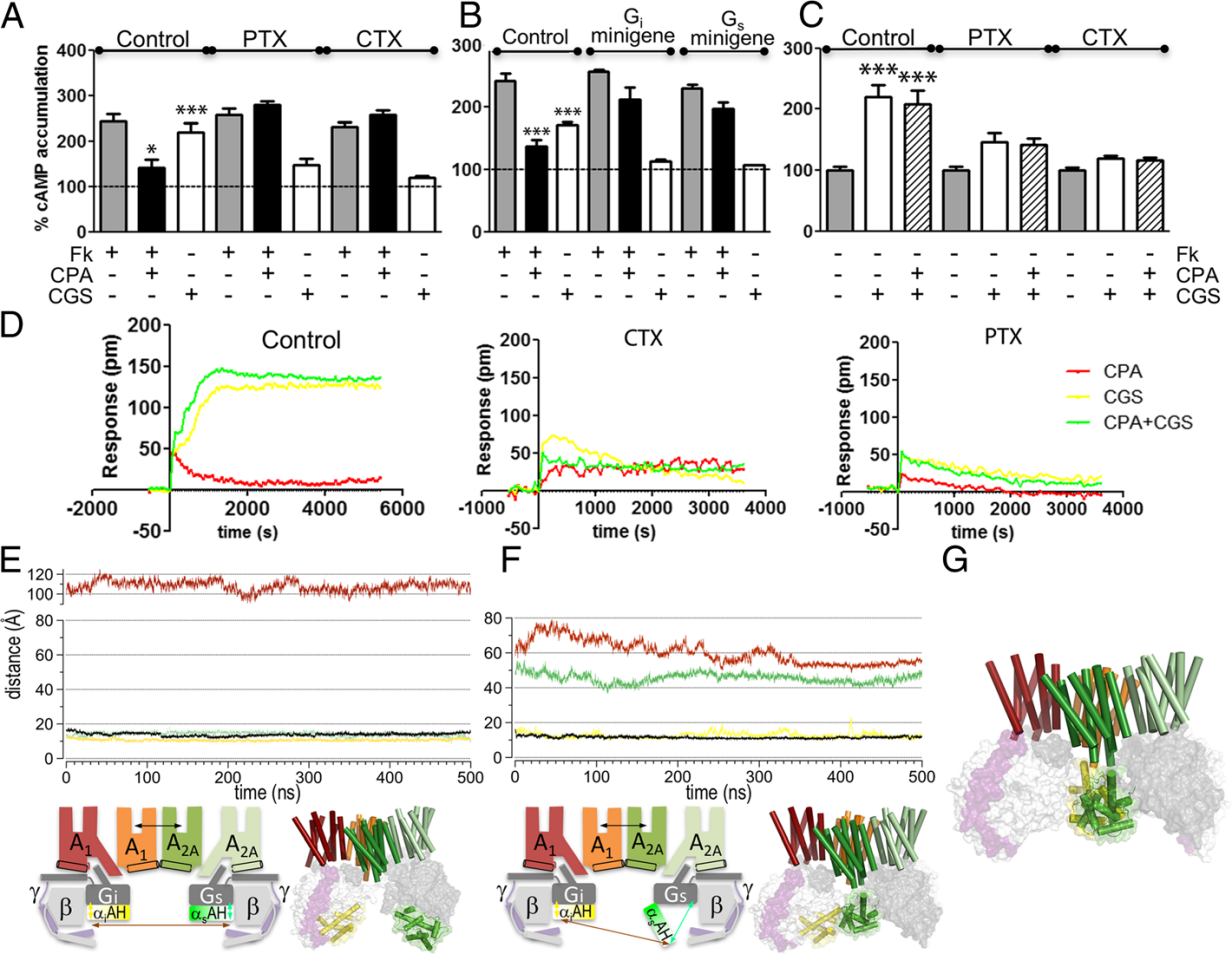
Receptor signaling through the A_1_-A_2A_Het. Increases in cAMP percentage accumulation with respect to Fk-stimulated (**a**, **b**) or unstimulated (**c**) cells. A_1_-A_2A_Het-expressed cells pre-treated with medium, PTX (10 ng/mL overnight) or CTX (100 ng/mL for 1 h) before adding medium, forskolin (Fk, 0.5 μM), CPA (100 nM) plus/minus forskolin, CGS-21680 (100 nM) plus/minus forskolin, or CPA + CGS-21680. **b** Same assays in the absence or presence of 0.5 μg of cDNA corresponding to G_i_- or G_s_-α-subunit-related minigenes. Mean ± SEM (7 experiments/group). One-way ANOVA followed by Bonferroni’s post-hoc test in panels **a**, **b** showed a significant effect over basal in samples treated with CGS-21680 or over forskolin in samples treated with CPA; in panel **c**, a significant effect is seen over basal (*P < 0.05, ***P < 0.001). **d** The dynamic mass redistribution analysis was plotted as pm shifts versus time (Representative experiment, performed in triplicate). **e**, **f** Distances between the Cα atoms of Arg90 (α_i_AH domain) and Glu238 (Ras domain) of G_i_ (in yellow), Asn112 (α_s_AH) and Asn261 (Ras) of G_s_ (green), Arg90 (α_i_AH) and Asn112 (α_s_AH) (dark red), and between the center of masses of the binding sites of the G_i_-unbound A_1_R and G_s_-unbound A_2A_R protomers (black) obtained from two independent molecular dynamics (MD) simulations of A_1_-A_2A_Het in complex with G_i_ and G_s_ in which α_i_AH was modelled in the closed conformation (Additional file 1: Figure S6C) and α_s_AH was modelled in closed (**e**) or open (**f**) conformation. The computed distances are depicted as double arrows in the adjacent schematic representations. Representative snapshots of the models are shown. Code: G_i_-bound A_1_R/red, G_i_-unbound A_1_R/orange, G_s_-bound A_2A_R/light green, G_s_-unbound A_2A_R/dark green, α, β, and γ of G_i_/G_s_ in dark gray/light gray/purple, respectively, TM4/light blue, TM5/gray, α-helical α_i_AH/green, and α_s_AH/yellow. **g** MD simulations could not be performed for open conformations of α_s_AH and α_i_AH due to steric clash

Next, we tested whether the A_1_-A_2A_Het can signal through G_s_- and G_i_-dependent pathways by measuring cAMP levels in cells expressing both A_1_R and A_2A_R. The A_1_R-selective agonist CPA (100 nM, a concentration producing maximal effect), which was unable to modify cAMP levels in the absence of forskolin (Additional file 1: Figure S2A), decreased forskolin-induced cAMP due to its G_i_ coupling, and the A_2A_R-selective agonist CGS21680 (100 nM, a concentration producing maximal effect) increased cAMP due to a G_s_ coupling (Fig. 3a, control), indicating that both receptors signal via their cognate G protein. We performed the same experiments in cells treated with pertussis (PTX) or cholera (CTX) toxins, which impair G_i_- and G_s_-mediated signaling, respectively, and in cells transfected with minigenes that encode for peptides blocking the interaction of the receptor with the α subunits of G_i_ or G_s_ [8]. As expected, we observed blockade of CPA-induced cAMP decrease by either PTX (Fig. 3a) or the G_i_-specific minigene (Fig. 3b), and blockade of CGS21680-induced cAMP increase by CTX (Fig. 3a) or the G_s_-specific minigene (Fig. 3b). Strikingly, PTX or G_i_-specific minigene (blocking G_i_-receptor interaction) also blocked the CGS21680-induced cAMP increase (Fig. 3a, b). Moreover, CTX or the G_s_-specific minigene (blocking G_s_-receptor interaction) also blocked the CPA-induced cAMP decrease (Fig. 3a, b). Control experiments using these agonists in cells expressing only A_1_R or A_2A_R did not show any crossover effect with either toxins or minigenes (Additional file 1: Figures S2B, C, E, F). These results demonstrate that both A_1_R- and A_2A_R-mediated signaling in the A_1_-A_2A_Het are dependent on the functional integrity of both G_i_ and G_s_ proteins. According to this, we observed by BRET experiments that the A_2A_R agonist-induced interaction between A_1_-A_2A_Het and G_s_ protein diminished in cells pre-treated with PTX (Additional file 1: Figure S1D). We hypothesize that this cross-communication could depend on the ability of α subunits of G_i_ and G_s_ coupled to the A_1_-A_2A_Het to establish mutual interactions (see below).

To further test for a cross-communication between G proteins in the G_s_-G_i_-heterotetramer signaling unit, we resolved the real-time signaling signature by using a label-free method, based on optical detection of dynamic changes in cellular density following receptor activation [9]. The magnitude of the signaling by CPA or by CGS 21680 significantly decreased when cells co-expressing both receptors were pre-treated with either PTX or CTX (Fig. 3d). This phenomenon was not observed in cells expressing only A_1_R (Additional file 1: Figure S2G) or A_2A_R (Additional file 1: Figure S2H). Again, these results indicate the simultaneous coupling of interacting G_s_ and G_i_ proteins within the A_1_-A_2A_Het.

Simultaneous activation of both A_1_R and A_2A_R with CPA and CGS21680 increased cAMP to similar levels to those obtained with CGS21680 alone and the signal of co-activated receptors was inhibited by both PTX and CTX (Fig. 3c). Therefore, A_1_R agonist was able to decrease forskolin-induced cAMP (Fig. 3a, b) and yet was unable to decrease A_2A_R-mediated increases of cAMP (Fig. 3c). Consequently, when both receptors are co-activated in the heterotetramer, only the A_2A_R-mediated, but not the A_1_R-mediated signaling occurs. This finding was confirmed in label-free experiments, showing that receptor co-activation with CPA and CGS 21680 did not increase the time-response curve with respect to the activation with CGS 21680 alone (Fig. 3d green and yellow lines, respectively).

It has been shown that the mechanism for receptor-catalyzed nucleotide exchange in G proteins involves a large-scale opening of the α-helical domain (αAH) of the α-subunit, from the Ras domain, allowing GDP to freely dissociate [10–13]. Notably, our proposed model of the A_1_-A_2A_Het positions the α_i_AH and α_s_AH domains facing each other (Fig. 3e). The fact that both G_s_- and G_i_-specific toxins and G_s_- and G_i_-specific minigenes affect both G_s_- and G_i_-mediated coupling in the A_1_-A_2A_Het suggests that the proposed large-scale conformational changes of αAH domains is mutually dependent. We used molecular dynamics (MD) simulations of the A_1_-A_2A_Het in complex with G_s_ and G_i_ to evaluate intermolecular distances between the α_s_AH and α_i_AH domains when α_i_AH is in the closed conformation and α_s_AH is either in the open (Fig. 3e) or in the closed conformation (Fig. 3ef). In a previous report, double electron–electron resonance (DEER) distance distributions between spin labels attached to Arg90 (α_i_AH domain) and Glu238 (Ras domain) of G_i_ (the distance between Cα atoms is termed d[Arg90α_i_-Glu238α_i_] in the manuscript) or Asn112 (α_s_AH) and Asn261 (Ras) of G_s_ (d[Asn112α_s_-Asn261α_s_]) permitted to faithfully monitor the equilibrium within the open (distance of ~40 Å) and closed (~20 Å) conformation of the αAH domain [13]. Here, we measured the intermolecular distance between the α_s_AH and α_i_AH domains using Cα atoms of Arg90 of α_i_ and Asn112 of α_s_ (d[Arg90α_i_-Asn112α_s_]). This d[Arg90α_i_-Asn112α_s_] intermolecular distance between α_i_AH in the closed conformation (d[Arg90α_i_-Glu238α_i_]: 11 Å, yellow line in Fig. 3e) and α_s_AH in the closed conformation (d[Asn112α_s_-Asn261α_s_]: 14 Å, green line in Fig. 3e) has an average value of 108 Å for inactive A_1_-A_2A_Het (Fig. 3e, dark red line). Activation of A_2A_R would trigger the opening of α_s_AH (d[Asn112α_s_-Asn261α_s_]: 52 Å; Fig. 3f, green line), necessary for GDP/GTP exchange, decreasing the d[Arg90α_i_-Asn112α_s_] distance between α_i_AH and α_s_AH to 60 Å (Fig. 3f, dark red line). Although the results are based on a single trajectory, it is unlikely that additional replicates would change, in a significant manner, the distances reported from the simulations. Moreover, the differences between the distances are so substantial that results from more simulations would not have a significant impact. We hypothesize that a similar change occurs with activation of A_1_R. This indicates that both receptors can signal via their cognate G protein by opening their αAH domain. However, in the compact rhombus-shaped A_1_-A_2A_Het model, simultaneous opening of both αAH domains (co-activation with CPA and CGS 21680) would not be possible due to a steric clash in such open conformations (Fig. 3g). Due to this steric clash, MD simulations of this open α_i_AH-open α_s_AH conformation in the absence of interference peptides (see below) cannot be performed.

### Altering the heteromer interface of A_1_-A_2A_Het enables simultaneous G_i_ and G_s_ signaling

**Fig. 4:**
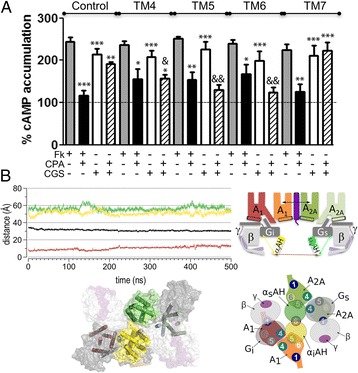
Effect of interference peptides on receptor signaling. **a** cAMP production was determined in HEK-293 T cells transfected with 0.4 μg of A_1_R and A_2A_R cDNAs. Cells were treated for 4 h with medium (control) or with 4 μM A_2A_R TM synthetic peptides (TM1 to TM7, see Methods). Cells were unstimulated (basal, dotted line) or stimulated with forskolin (Fk, 0.5 μM, gray bars), with forskolin and the A_1_R agonist CPA (100 nM, black bars), the A_2A_R agonist CGS-21680 (100 nM, white bars), or with CPA and CGS-21680 (striped bars). Increases in cAMP percentage accumulation in relation to unstimulated cells. Mean ± SEM (7 experiments/condition). One-way ANOVA followed by Bonferroni’s post-hoc test showed a significant effect over basal in samples treated with CGS-21680 or CGS-21680 plus CPA, or over forskolin in samples treated with CPA (*P < 0.05, **P < 0.01, ***P < 0.001). One-way ANOVA followed by Bonferroni’s post-hoc test showed a significant effect over control in the absence of peptide (&*P* < 0.05, &&*P* < 0.01). **b** Intermolecular distances (depicted as double arrows in the adjacent schematic representation) were obtained from MD simulations of A_1_-A_2A_Het in complex with G_i_ and G_s_ (α_i_AH and α_s_AH were modeled in the open conformation, see Additional file 1: Figure S2B) in the presence of the TAT-fused TM6 peptide, which alters the heteromer interface between A_1_R and A_2A_R. A representative snapshot of the molecular model is shown, viewed from the intracellular site. The TAT-TM6 peptide is shown in purple, whereas the color code of the depicted proteins is as in Fig. 3

Next, we investigated whether the correct formation of the A_1_-A_2A_Het is a necessary condition for the crosstalk between the G_s_- and G_i_-signaling units using the interference peptides (TM4, TM5 and TM6 of A_2A_R, which alter receptor heterodimerization, and TM7 as a negative control). Remarkably, pretreatment of cells expressing A_1_-A_2A_Het with the interference peptides did not change receptor signaling when only one receptor is activated (Fig. 4a). Interestingly, in the presence of TM4, TM5 and TM6 peptides, simultaneous activation of both A_1_R and A_2A_R with CPA and CGS21680, respectively, allows CPA to decrease CGS21680-stimulated cAMP (Fig. 4a), in contrast to experiments in the absence of either interference peptides (Fig. 4a, control) or TM7 used as a negative control (Fig. 4a). Moreover, this decrease in cAMP accumulation in the CPA/CGS co-stimulated condition is mediated by activation of the A_1_R/G_i_ pathway as, in the presence of TM peptides, a selective A_1_R antagonist or the treatment with PTX blocks the CPA-induced effect (Additional file 1: Figure S2D). Thus, modification of the quaternary structure of the A_1_-A_2A_Het with peptides that penetrate within the heteromer interface abolishes inhibition of A_1_R by A_2A_R in the G_s_-G_i_-heterotetramer signaling unit. These experimental results suggest that synthetic peptides inserted between A_1_R and A_2A_R protomers, which are not able to disrupt the heteromer as seen by PLA (Fig. 2), increase the distance between G_i_ and G_s_. This would allow the simultaneous opening of α_i_AH and α_s_AH domains for GDP dissociation. In order to verify this hypothesis, we modeled the A_1_-A_2A_Het with the TAT-fused peptide TM6 altering the heteromer interface between A_1_R and A_2A_R, in complex with G_s_ (open α_s_AH, d[Asn112α_s_-Asn261α_s_]: 56 Å; Fig. 4b, green line) and G_i_ (open α_i_AH, d[Arg90α_i_-Glu238α_i_]: 52 Å; Fig. 4b, yellow line). Due to the insertion of TM6, the distance between the binding site of A_1_R and A_2A_R increases by 17 Å, from 14 Å in the absence of TM6 (Fig. 3e, f, black line) to 31 Å in the presence of TM6 (Fig. 4b, black line). This increase in the distance between heteromers also moves the intracellular α_i_AH and α_s_AH domains apart, thus permitting their simultaneous opening (d[Arg90α_i_-Asn112α_s_]: 10 Å; Fig. 4b, dark red line) for GDP/GTP exchange.

### A_1_-A_2A_Het as an adenosine concentration-sensing device

**Fig. 5:**
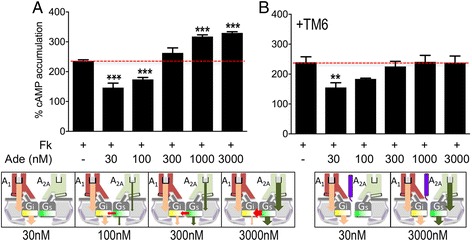
A_1_-A_2A_Het as an adenosine concentration-sensing device. A_1_-A_2A_Het-expressed cells were treated for 4 h with medium (**a**) or with 4 μM of the synthetic A_2A_R TM6 peptide (**b**). Cells were stimulated with forskolin (Fk, 0.5 μM, red broken line) and adenosine at increasing concentrations (30–3000 nM, black bars). cAMP levels were expressed as percentage over unstimulated cells (basal, 100%). Mean ± SEM of (7 experiments/condition). One-way ANOVA followed by a Dunnett’s multiple comparison tests showed statistical differences relative to cells stimulated only with forskolin (**P < 0.01, ***P < 0.001). Bottom panels show schemes that may provide an explanation of the results obtained at each adenosine concentration. (1) The higher affinity of adenosine for A_1_R than for A_2A_R is illustrated by the size of the black lines at the binding site (adenosine is shown as gray rectangles). (2) Adenosine-induced A_1_R and A_2A_R activation are depicted as arrows in pink and green, respectively, starting at the binding site of each receptor. (3) A_1_R-induced G_i_ activation and A_2A_R-induced G_s_ activation, with the corresponding decrease/increase of cAMP, are depicted as arrows in pink and green, respectively. The inhibitory effect of G_s_ on G_i_-mediated signaling is shown as a red arrow. Width of arrows illustrates the magnitude of receptor or G protein activation or cross-talk. High adenosine concentrations increase the A_2A_R binding (gray rectangle), the adenosine-induced A_2A_R activation, the A_2A_R-induced G_S_ activation (green arrows) and the cross-talk among G proteins (red arrow), while decreasing the A_1_R-induced G_i_ activation (pink arrow) due to the cross-talk. In the presence of TM6 (in purple) the cross-talk among G proteins is lost, enabling simultaneous A_1_R-induced G_i_ activation (pink arrow) and A_2A_R-induced G_S_ activation (green arrow)

In order to illustrate the molecular device allowing adenosine to signal by one or the other receptor [4], we measured cAMP levels at increasing concentrations of adenosine in cells expressing the A_1_-A_2A_Het (Fig. 5a). Due to the higher affinity for the hormone, adenosine at a low concentration (30 nM) binds predominantly to A_1_R and engages a G_i_-mediated signaling, which significantly decreases forskolin-induced cAMP accumulation. At higher concentrations, adenosine progressively binds to A_2A_R, which engages a G_s_-mediated signaling. At high adenosine concentrations, full occupancy of both A_1_R and A_2A_R leads to marked increases in cAMP levels compatible with G_s_ activation and blockade of G_i_, as depicted in the schemes of Fig. 5a. In these conditions, full active A_2A_R can increase cAMP over the forskolin-induced levels whilst the progressive blockade of A_1_R by A_2A_R cannot reduce cAMP accumulations. To demonstrate such blockade of A_1_R actions by A_2A_R, we performed the experiments in the presence of a peptide (A_2A_R TM6) that inserts into the heteromer interface (Fig. 5b). In the presence of the peptide, the device lost its concentration-sensing properties. In fact, high adenosine concentrations, in which both receptors are fully occupied and functional, led to a null response, i.e., the A_2A_R-mediated increase in forskolin-stimulated cAMP is counteracted by a similar G_i_-mediated decrease of cAMP. Upon heteromer structure alteration by TM6, the A_2A_R becomes unable to block A_1_R-mediated signaling.

### Recruitment of β-arrestin-2 by the A_1_-A_2A_Het

**Fig. 6:**
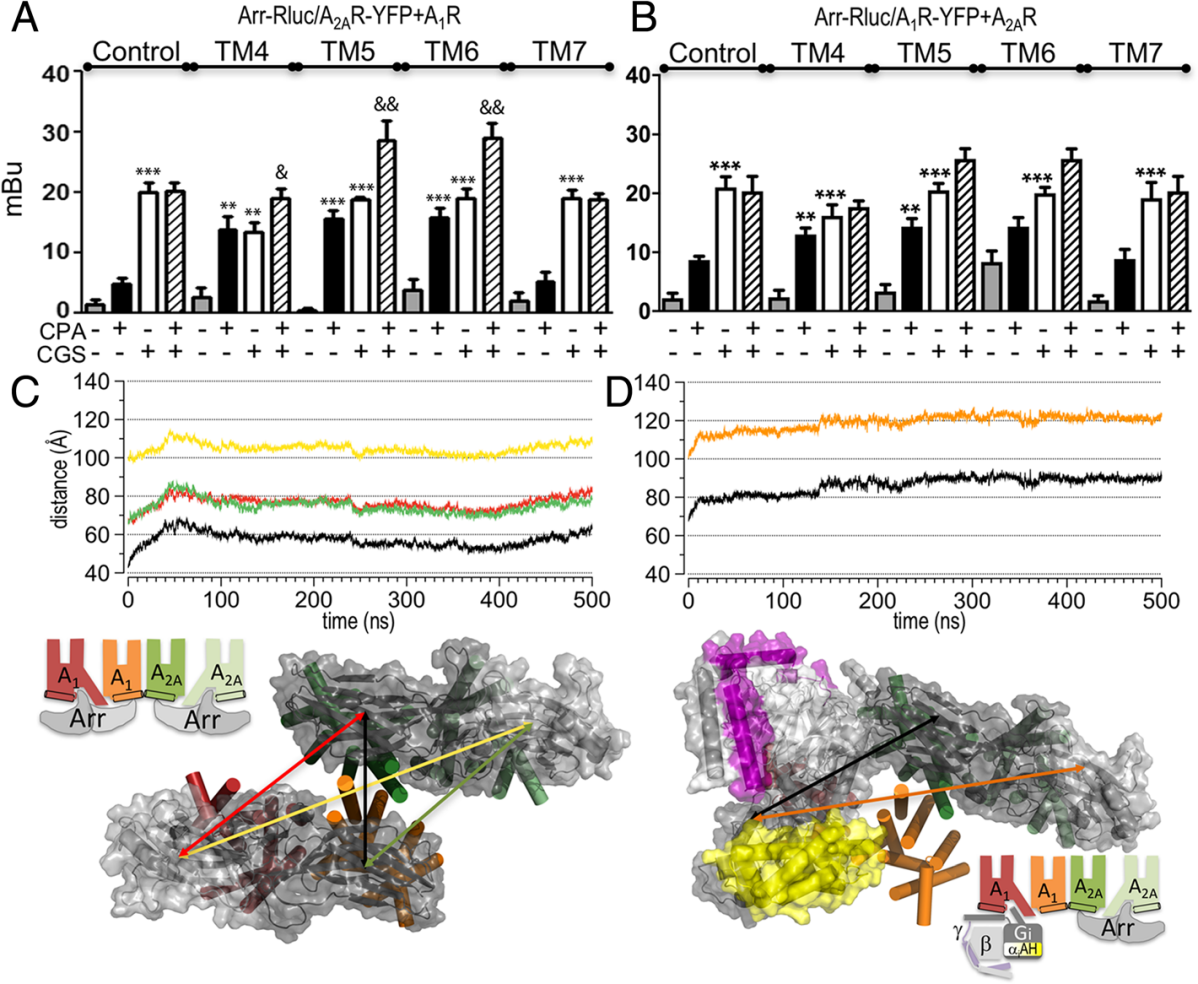
Effect of interference peptides on recruitment of β-arrestin-2. **a**, **b** Receptor agonist-induced β-arrestin-2 recruitment was measured by BRET. HEK-293 T cells were transfected with the cDNAs for β-arrestin-2-Rluc (Arr-Rluc, 0.5 μg cDNA) and either A_2A_-YFP (0.4 μg cDNA) and A_1_R (0.4 μg cDNA) (**a**) or A_1_-YFP (0.4 μg cDNA) and A_2A_R (0.4 μg cDNA) (**b**). Cells were untreated (control) or treated for 4 h with 4 μM A_2A_R TAT-TM synthetic peptides (TM4–7, see Methods) before addition of medium (basal, gray bars) or 100 nM of either the A_1_R agonist CPA (black bars), the A_2A_R agonist CGS-21680 (CGS, white bars), or both (striped bars). Positive BRET was expressed as milli-BRET units (see Methods). Mean ± SEM (7 experiments/condition). One-way ANOVA followed by Bonferroni’s post-hoc test showed a significant effect over basal in samples treated with CGS-21680 or over forskolin in samples treated with CPA (*P < 0.05, **P < 0.01, ***P < 0.001). One-way ANOVA followed by Bonferroni’s post-hoc test showed a significant effect of CPA + CGS-21680 over CGS-21680 treatments (&*P* < 0.05, &&*P* < 0.01). **c** Intermolecular distances between the center of masses of the N- and C-domains of the A_1_R-bound arrestin and of the A_2A_R-bound arrestin obtained from molecular dynamics (MD) simulations of A_1_-A_2A_Het in complex with two molecules of β-arrestin-2. **d** Intermolecular distances between the center of mass of the N- and C-domains of the A_2A_R-bound arrestin and the Cα atom of Glu238 (RAS domain) of G_i_ obtained from MD simulations of A_1_-A_2A_Het in complex with G_i_ bound to A_1_R and β-arrestin-2 bound to A_2A_R. These intermolecular distances are depicted as double arrows in the adjacent representative snapshots of the molecular models. Arrestin is shown in gray, whereas the color code of the depicted proteins is as in Fig. 3

We used BRET assays to detect the interaction between a protomer and β-arrestin-2. Thus, cells were transfected with cDNAs of β-arrestin-2 fused to Rluc (Arr-Rluc) as the BRET donor and A_1_R or A_2A_R fused to YFP (A_1_R-YFP, A_2A_R-YFP) as the BRET acceptor. Control experiments in cells expressing only A_1_R-YFP or A_2A_R-YFP and Arr-Rluc show the ability of both receptors to recruit β-arrestin-2 (Additional file 1: Figure S3A) and the selectivity of each agonist (Additional file 1: Figure S3B). Similar experiments in cells additionally expressing non-fused A_2A_R (Arr-Rluc/A_1_R-YFP + A_2A_R) or non-fused A_1_R (Arr-Rluc/A_2A_R-YFP + A_1_R) were performed (Additional file 1: Figure S3B). Interestingly, in cells expressing Arr-Rluc, A_2A_R-YFP and non-fused A_1_R (control in Fig. 6a and Additional file 1: Figure S3B) or Arr-Rluc, A_1_R-YFP and non-fused A_2A_R (control in Fig. 6b and Additional file 1: Figure S3B), a similar degree of BRET was induced by CGS-21680 (white bars) or by CGS-21680 plus CPA (striped bars). This suggests that agonist binding to A_2A_R inhibits the CPA ability to stimulate β-arrestin-2 recruitment to A_1_R. In order to rationalize these results, we have used the recent crystal structure of rhodopsin bound to visual arrestin-1 [14] to model the A_1_-A_2A_Het in complex with β-arrestin-2. The finger loop of arrestin, which adopts a short α-helix, is inserted into the intracellular cavity of the external protomer, whereas the C-domain of arrestin points towards the internal protomer of the homodimer. Figure 6c shows key intermolecular distances between the center of mass of the N- and C-domains of two arrestin molecules bound to A_1_R and A_2A_R obtained from MD simulations. These data suggest that the A_1_-A_2A_Het quaternary structure permits the binding of two arrestin molecules to the external protomers of both A_1_R and A_2A_R, similarly to the simultaneous binding of G_i_ and G_s_ to the heterotetramer. Moreover, similar simulations of A_1_-A_2A_Het in complex with G_i_ and β-arrestin-2 (Fig. 6d) show no steric clashes between G_i_ (bound to A_1_R) and arrestin (bound to A_2A_R). These results suggest that sustained activation of G_s_ (G_βγ_ moving away from G_αs_ to facilitate the interaction of G_αs_ with the catalytic domain of adenylate cyclase) by agonist binding to A_2A_R enables β-arrestin-2 recruitment to A_2A_R. As stated above, within the A_1_-A_2A_Het, CPA cannot activate G_i_ in the presence of the A_2A_R agonist CGS-21680 (Fig. 3) and, consequently, CPA does not trigger additional β-arrestin-2 recruitment to A_1_R (control in Figs. 6a, b and Additional file 1: Figure S3B).

Using the TAT-fused synthetic peptides we investigated whether the quaternary structure of the A_1_-A_2A_Het determines its putative selective A_2A_R-dependent β-arrestin-2 recruitment. As a negative control, we first corroborated that TM4, TM5, and TM6 peptides of A_2A_R do not interfere with A_1_R-mediated signaling (Additional file 1: Figure S3C). Pretreatment of cells expressing Arr-Rluc, A_2A_R-YFP and non-fused A_1_R (Fig. 6a), or Arr-Rluc, A_1_R-YFP and non-fused A_2A_R (Fig. 6b) with TM4, TM5, and TM6 peptides, but not in the absence of peptides (control) or with the TM7 peptide (negative control), allowed the detection of positive BRET (recruitment of β-arrestin-2) not only when cells were treated with the A_2A_R-selective agonist CGS-21680 (white bars), but also when treated with the A_1_R-selective agonist CPA (black bars) (Figs. 6a, b). Importantly, when cells expressing Arr-Rluc, A_2A_R-YFP, and non-fused A_1_R were co-activated by CPA and CGS-21680 (striped bars), BRET measurement in the presence of TM4, TM5, or TM6 peptides, but neither in the absence of peptides nor in the presence of TM7 peptide, significantly increased relative to the values obtained by the action of a single agonist (Fig. 6a). The trend is similar in cells expressing Arr-Rluc, A_1_R-YFP, and non-fused A_2A_R, but not statistically significant (Fig. 6b). These results indicate that alteration of the A_1_R-A_2A_R heteromer interface within the A_1_-A_2A_Het allows simultaneous recruitment of β-arrestin-2 to A_1_R and A_2A_R when both receptors are activated. Interference peptides abolish cross-communication of G proteins, permitting CPA to activate G_i_ (G_βγ_ moving away from G_αi_) and recruitment of β-arrestin-2 to A_1_R, as well as G_s_ activation by CGS-21680 (G_βγ_ moving away from G_αs_) and simultaneous recruitment of β-arrestin-2 to A_2A_R.

### The C-terminal domain of A_2A_R is responsible for the dominant A_2A_R-mediated signaling

**Fig. 7:**
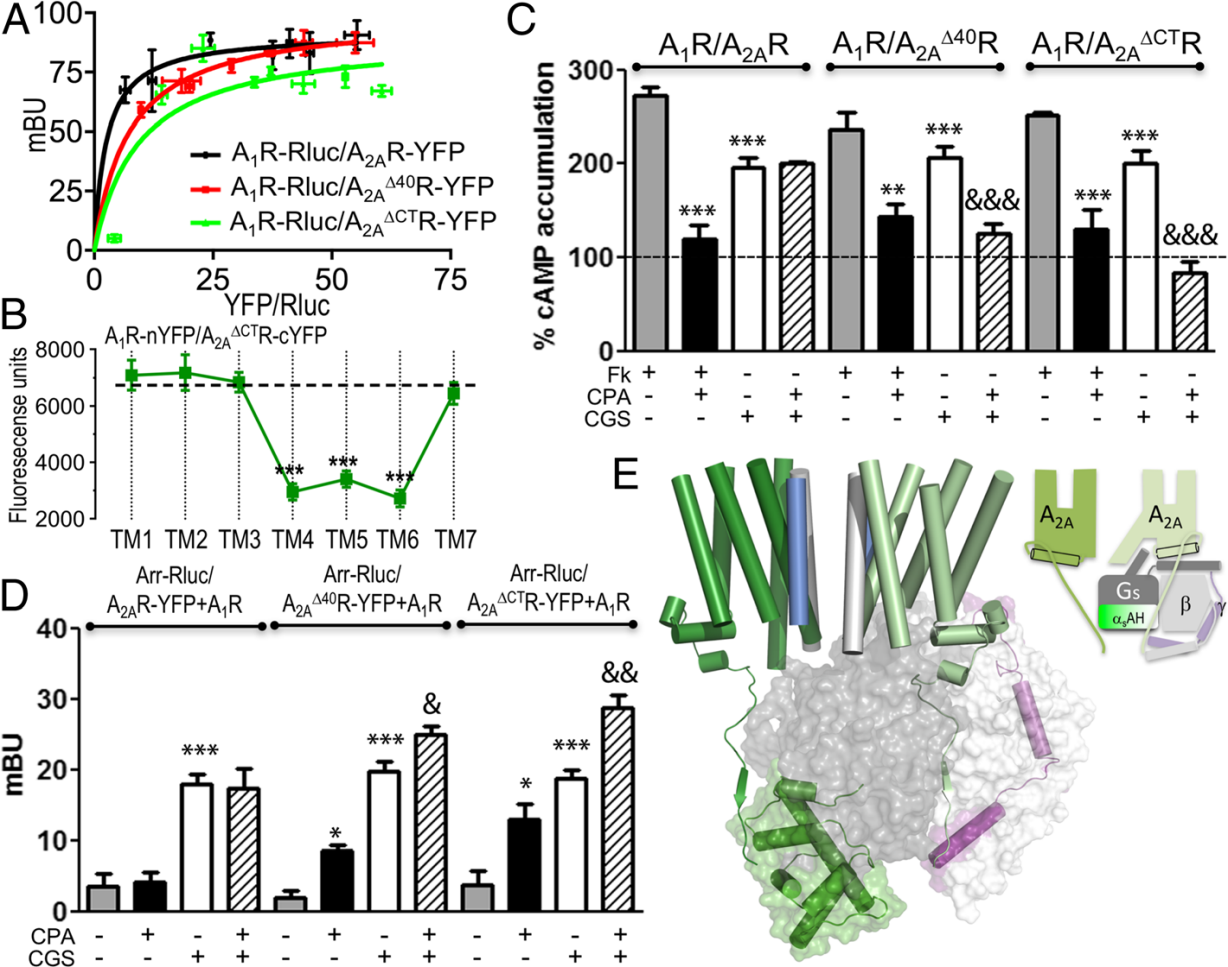
Influence of A_2A_R C-terminal domain over signaling properties of A_1_-A_2A_Hets. **a** BRET in cells expressing constant A_1_-Rluc amount (0.4 μg cDNA) and increasing (0.1–0.7 μg cDNA) amounts of A_2A_^Δ40^R-YFP or A_2A_^ΔCT^R-YFP. Mean of milli-BRET units ± SEM (*n* = 7). **b** BiFC assays (fluorescence measured at 530 nm) were performed in cells expressing (1 μg cDNA) A_1_R-nYFP and A_2A_^ΔCT^R-cYFP and pre-treated for 4 h with medium or 4 μM A_2A_R-TM peptides (TM1–7). Mean ± SEM (13 experiments/treatment). One-way ANOVA followed by a Dunnett’s multiple comparison test showed a significant fluorescence decrease over control values (***P < 0.001). **c** HEK-293 T cells expressing A_1_R (0.4 μg cDNA) and A_2A_R (0.3 μg cDNA), A_2A_^Δ40^R (0.3 μg cDNA), or A_2A_^ΔCT^R (0.3 μg cDNA) were unstimulated (basal, dotted line) or stimulated with forskolin (Fk, 0.5 μM, gray bars), with forskolin and CPA (100 nM, black bars), CGS-21680 (CGS, 100 nM, white bars), or with CPA + CGS-21680 (striped bars). cAMP percentage accumulation over unstimulated cells. Mean ± SEM (7 experiments/group). One-way ANOVA followed by Bonferroni’s post-hoc test: significant effect over basal in CGS-21680-stimulated samples or over forskolin-stimulated cells (**P < 0.01, ***P < 0.001) or CPA + CGS-21680 over CGS-21680-stimulated cells (&&&*P* < 0.001). **d** HEK-293 T cells expressing β-arrestin-2-Rluc (Arr-Rluc, 0.5 μg cDNA), A_1_-YFP (0.4 μg) and A_2A_R (0.3 μg), A_2A_^Δ40^R (0.3 μg), or A_2A_^ΔCT^R (0.3 μg). Cells stimulated with agonists as indicated. Mean ± SEM (7 experiments/condition). One-way ANOVA followed by the Bonferroni’s post-hoc test: significant differences over unstimulated cells (*P < 0.05, ***P < 0.001) or CPA-CGS-21680 over CGS-21680-stimulated cells (&*P* < 0.05, &&*P* < 0.01). **e** Molecular model of the A_2A_R homodimer in complex with G_s_. TMs involved in homodimerization: TM4/light blue and TM5/gray; color code of proteins is as in Fig. 3. C-tail of G_s_α-subunit-unbound A_2A_R protomer is near α_s_AH (shown in closed conformation)

Despite the apparent structural symmetry of the GPCR/G protein macromolecular complex, a major difference is the length of the intracellular C-terminal domain of adenosine receptors (16 amino acids in A_1_R versus 102 in A_2A_R). The short C-terminal tail of the A_1_R does not have any known specific function, while the C-terminus of A_2A_R, albeit dispensable for ligand binding [15], dimerization [16], and agonist induced cAMP signaling [17], influences constitutive signaling [18]. Due to the shorter C-terminus of A_1_R and the proposed orientation of the C-tail of A_2A_R toward α_s_AH (see Additional file 1: Figure S4a for details), as well as the proposed role of the C-terminal tail in downstream signaling cascade activation [19], we speculated that the C-terminus of A_2A_R could modulate the prevailing G_s_-mediated signaling upon A_1_R and A_2A_R co-activation. To test this hypothesis, we engineered two A_2A_R mutants, one lacking most of the C-terminal end (A_2A_^ΔCT^R) and another lacking the last 40 amino acids (A_2A_^Δ40^R). First, we tested whether these truncated versions of A_2A_R could form heteromers with A_1_R. We observed similar BRET saturation curves in HEK-293 T cells expressing a constant amount of A_1_R-Rluc cDNA and increasing amounts of either A_2A_R-YFP, A_2A_^Δ40^R-YFP, or A_2A_^ΔCT^R-YFP, indicating that A_2A_^Δ40^R and A_2A_^ΔCT^R form heteromers with A_1_R (Fig. 7a; BRET_max_ in mU: 91 ± 3 A_2A_R, 99 ± 3 A_2A_^Δ40^R, and 90 ± 8 A_2A_^ΔCT^R). Heteromers were also detected by BiFC assays in HEK-293 T cells transfected with cDNAs for A_1_R-nYFP and A_2A_^ΔCT^R-cYFP (Fig. 7b, dashed line). In these cells, fluorescence was reduced in the presence of TM4, TM5, and TM6 peptides of A_2A_R (Fig. 7b). Thus, heteromerization of A_2A_^ΔCT^R with A_1_R occurs via the TM5/6 interface, similarly to the interaction of A_2A_R with A_1_R.

We measured cAMP production in cells expressing A_1_R and wild-type or truncated A_2A_R receptors (Fig. 7c). Truncated A_2A_R were able to signal as wild-type receptors. Interestingly, the dominant G_s_-mediated signaling when A_1_R and A_2A_R were co-activated decreased progressively with the shortening of the A_2A_R C-tail (Fig. 7c, striped bars). In fact, CPA inhibited CGS-21680-induced cAMP accumulation when truncated receptors were expressed, showing that, in these heteromers, A_1_R were functional (Additional file 1: Figure S5). Figure 7e shows a detailed view of the orientation of the C-tail (102 amino acids, Gln311-Ser412) of both A_2A_R protomers in the A_1_-A_2A_Het, which was modeled as suggested for the OXER [20], together with the structure of β-arrestin-2 in complex with V2 vasopressin receptor [21]. It is important to note that the exact conformation of the A_2A_R C-tail cannot unambiguously be determined, thus, we only predict its orientation as explained in detail in Additional file 1: Figure S4. The fact that the C-tail of the α_s_-unbound A_2A_R protomer points toward the α_s_AH domain suggests that this C-tail is influencing the conformational changes required to open the α_s_AH, and thus controlling the balance between G_s_ and G_i_ activation. Next, we measured β-arrestin-2 recruitment by BRET assays in cells expressing A_1_R and wild-type or truncated A_2A_R receptors. In cells expressing non-fused A_1_R, Arr-Rluc and A_2A_R-YFP, A_2A_^Δ40^R-YFP, or A_2A_^ΔCT^R-YFP, the A_1_R agonist CPA could increase BRET values only when the heteromer is formed with A_2A_R-truncated receptors. In these conditions, co-activation with CPA and CGS-21860 induced a BRET increase higher than the one obtained with CGS-21680 alone (Fig. 7d). These results indicate that the selective A_2A_R-dependent β-arrestin-2 recruitment in the A_1_-A_2A_Het decreases progressively with the shortening of the A_2A_R C-tail (Fig. 7d).

## Discussion

As previously reviewed [2, 3, 22], the intercommunication between protomers of a GPCR heteromer can be observed at the level of agonist binding, ligand-induced cross-conformational changes between receptor protomers, and the binding of GPCR-associated proteins, including heterotrimeric G proteins and β-arrestins. The intercommunication between protomers is a consequence of a defined quaternary structure that is responsible for the specific functional characteristics of the heteromer. For GPCR heteromers, such as A_1_-A_2A_Het, constituted by receptors sensing the same hormone but producing opposite signaling effects, it is not obvious how a defined quaternary structure achieves this dual behavior. A_1_-A_2A_Het acts as a concentration-sensing device that allows adenosine to signal by one or the other coupled G protein (G_s_ or G_i_) to fine-tune modulate the release of neurotransmitters from presynaptic terminals. In the present study, we solved this question by discovering a new mechanism of signal transduction, a cross-communication between G_i_ and G_s_ in the A_1_-A_2A_Het guided by the A_2A_R C-terminal domain.

We have shown that cross-communication between G_i_ and G_s_ proteins involves the formation of a GPCR heterotetramer (i.e., one homodimer of A_1_R and one of A_2A_R) that has a 2:2:1:1 (A_2A_R:A_1_R:G_s_:G_i_) stoichiometry. From our data, it is deduced that the cross-talk between G_i_ and G_s_ resides on the structural constraints surrounding the mechanism for GDP/GTP exchange, which involves the opening of the αAH domain of the α-subunit of any given G protein. We propose that cross-communication in the G_s_-G_i_-heterotetramer signaling unit is a property associated with a specific quaternary structure, the compact rhombus-shaped A_1_-A_2A_Het (the TM4/5 interface for homodimerization and the TM5/6 interface for heterodimerization), which positions the α_i_AH and α_s_AH domains in close proximity, making their conformational changes mutually dependent in a way that simultaneous opening of both αAH domains would not be possible due to a steric clash in such open conformations. Alterations of this quaternary structure of the A_1_-A_2A_Het by insertion of synthetic peptides between A_1_R and A_2A_R blocks this cross-communication without disrupting the heteromer and permits simultaneous activation of G_i_ and G_s_ in the heteromer_._ Since the cross-talk between G_i_ and G_s_ resides on the structural constraints imposed by defined TM interfaces in the heteromer, it is important to note that other heterotetramers, mainly those sensing different hormones and with a different quaternary structure, might not display this cross-communication among G proteins. Moreover, although, from a structural point of view, the A_1_-A_2A_Het is capable to recruit not only two G proteins but also two β-arrestins, the cross-talk between G_i_ and G_s_, in which G_s_ activation inhibits the simultaneous activation of G_i_, blocks A_1_R agonist-promoted arrestin recruitment. Alteration of the A_1_-A_2A_Het by insertion of synthetic peptides between A_1_R and A_2A_R facilitates simultaneous activation of G_i_ and G_s_ and the corresponding binding of two β-arrestins to A_1_R and A_2A_R. Our finding that G_i_ is dependent on G_s_-mediated signaling strengthens the conclusion that cross-talk across G proteins is a potentially important functional property of GPCR heteromers. Remarkably, when both receptors are co-activated in this heterotetramer, only A_2A_R-mediated, but not A_1_R-mediated signaling occurs. We show that the ability of blunting A_1_R-mediated signaling when G_s_ is engaged is dependent of the long C-terminus of the A_2A_R. In the absence of A_2A_R activation by agonists, or in the absence of the C-terminal domain of A_2A_R, the A_1_R-mediated signaling via G_i_ is totally functional. The most straightforward hypothesis is that the opening of α_s_AH parallels a movement of the C-tail to block the opening of α_i_AH.

Adenosinergic signaling in mammalians is important for energy and temperature homeostasis and for neuroregulation. Multiplicity of adenosine actions is due to a balance between the expression of specific receptors and producing/degrading enzymes and to the biological diversity due to a membrane network established by the interaction among purinergic receptors [23]. Ciruela et al. [4] first identified the occurrence of heteromers formed by A_1_R-G_i_- and A_2A_-G_s_-coupled adenosine receptors that participate in the regulation of glutamate release by neurons projecting from the cortex to the striatum. The same A_1_-A_2A_Het can be found in astrocytes modulating the transport of γ-amino butyric acid (GABA) [24]. Differently from the modulation of neuronal glutamate release, the A_1_R-G_i_-coupled receptor activates and the A_2A_R-G_s_-coupled receptor inhibits the modulation of GABA transport. Under conditions of high extracellular adenosine concentrations, such as hypoxic conditions [25], the nucleoside will bind to both the high (A_1_R) and the low (A_2A_R) affinity receptors in the heteromer, and the predominant A_2A_R-mediated signaling via G_s_ will result in counteraction of astrocytic GABA transport. Our results show that the asymmetric signaling is possible because the long C-terminus of A_2A_R blunts G_i_-mediated signaling. We have therefore elucidated the mechanism by which the A_1_-A_2A_Het functions as an adenosine concentration-sensing device that can promote even opposite signaling responses depending on the extracellular concentration of adenosine. The molecular mechanism involves the C-terminal domain of the activated G_s_-coupled A_2A_R, which hinders the activation of A_1_R coupled to G_i_.

## Conclusions

Using a convergent approach including biochemical, biophysical, cell biology, and molecular biology techniques, together with in silico molecular models, we here provide the mode of action of a membrane receptor complex that responds depending on the concentration of adenosine, a hormone and a neuroregulatory molecule. The concentration sensor is a heteromer composed of four adenosine receptors (two A_1_ and two A_2A_) and two G proteins (Gi and Gs). Despite Gi sits underneath the A_1_ receptor dimer and Gs sits underneath the A_2A_ receptor dimer, both G proteins do interact and are able to convey allosteric regulation depending on how the functional unit is activated. At low adenosine concentrations Gi is engaged via A1 activation without affecting/engaging Gs signaling. At higher concentrations Gs is engaged via A_2A_ activation, and this engagement blocks Gi-mediated signaling. The reason why a rhombus-shaped apparently symmetric structure results in asymmetric signaling is due to the long C-terminal tail of the A_2A_ receptor. In fact, both deletion of the C-terminal end or treatment with interfering peptides derived from the sequence of TM segments of the receptors impair allosteric cross-interaction between receptors and G proteins within the macromolecule, and the device loses its concentration sensing properties.

## Methods

### Cell culture and transient transfection

HEK-293 T cells were grown at 37 °C in in Dulbecco’s modified Eagle’s medium (DMEM) (Gibco) supplemented with 2 mM L-glutamine, 100 U/mL penicillin/streptomycin, and 5% (v/v) heat inactivated fetal bovine serum (all supplements were from Invitrogen, Paisley, Scotland, UK). Cells were transiently transfected with cDNA corresponding to receptors, fusion proteins, A_2A_R mutant constructs, or minigene vectors using polyethylenimine (Sigma-Aldrich, Cerdanyola del Vallés, Spain) as described elsewhere [7].

### Expression vectors, A_2A_R mutants and minigenes

Sequences encoding amino acid residues 1–155 or 155–238 of YFP-Venus protein, were subcloned in pcDNA3.1 to obtain the YFP Venus hemi-truncated proteins (nYFP and cYFP). The human cDNAs for A_2A_R, mutant A_2A_R, A_1_R, and Gi or Gs proteins cloned into pcDNA3.1, were amplified without their stop codons using sense and antisense primers harboring unique EcoRI and BamHI sites to subclone receptors in pcDNA3.1RLuc vector (p*RLuc*-N1 PerkinElmer, Wellesley, MA, USA) and EcoRI and KpnI to subclone receptors in pEYFP-N1 (enhanced yellow variant of GFP; Clontech, Heidelberg, Germany), pcDNA3.1-nVenus, or pcDNA3.1-cVenus vectors. The amplified fragments were subcloned to be in-frame with restriction sites of the corresponding vectors to give the plasmids that express receptors fused to RLuc, YFP, nYFP or cYFP on the C-terminal end (A_1_R-Rluc, A_2A_R-Rluc, Gi-RLuc, Gs-RLuc, A_1_R-YFP, A_2A_R-YFP, A_2A_^Δ40^R-YFP, A_2A_^ΔCT^R-YFP, A_1_R-nYFP, A_2A_-nYFP, and A_2A_-cYFP). Expression of constructs was tested by confocal microscopy and the receptor-fusion protein functionality by second messengers, ERK1/2 phosphorylation and cAMP production as described previously [4, 26–28]. Mutants with a deletion of aa 372 to aa 412 (A_2A_^Δ40^R) or aa 321 to aa 412 (A_2A_^ΔCT^R) on the C-terminal domain of A_2A_R were generated as previously described [29]. “Minigene” plasmid vectors are constructs designed to express relatively short polypeptide sequences following their transfection into mammalian cells. Here, we used minigene constructs encoding 11 amino acid residues from the C-terminus sequence of α subunit of G_i1/2_ or G_s_. The peptide coded by every minigene inhibits the coupling of the G (G_i1/2_ or G_s_) protein to the receptor and, consequently, it inhibits the G-protein-mediated cellular response, as previously described [8]. The cDNA encoding the last 11 amino acids of human G_α_ subunit corresponding to G_i1/2_ (IKNNLKDCGLF) or G_s_ (QRMHLRQYELL), inserted in a pcDNA 3.1 plasmid vector, was generously provided by Dr. Heidi Hamm.

### TAT-TM peptides

Peptides with the sequence of the TM of A_1_R and A_2A_R fused to the HIV TAT peptide (YGRKKRRQRRR) were used as oligomer-disrupting molecules (synthesized by Genemad Synthesis Inc. San Antonio, TX, USA). The cell-penetrating TAT peptide allows intracellular delivery of fused peptides [6]. The TAT-fused TM peptide can then be inserted effectively into the plasma membrane because of the penetration capacity of the TAT peptide and the hydrophobic property of the TM moiety [30]. To obtain the right orientation of the inserted peptide, the HIV-TAT peptide was fused to the C-terminus or to the N-terminus as indicated:

- MEYMVYFNFFVWVLPPLLLMVLIYLYGRKKRRQRRR for TM5 of A_1_R, RRRQRRKKRGYLALILFLFALSWLPLHILNCITLF for TM6 of A_1_R, ILTYIAIFLTHGNSAMNPIVYAFRIYGRKKRRQRRR for TM7 of A_1_R, VYITVELAIAVLAILGNVLVCWAVWYGRKKRRQRRR for TM1 of A_2A_R, YGRKKRRQRRRYFVVSLAAADIAVGVLAIPFAITI for TM2 of A_2A_R, LFIACFVLVLTQSSIFSLLAIAIYGRKKRRQRRR for TM3 of A_2A_R, YGRKKRRQRRRAKGIIAICWVLSFAIGLTPMLGW for TM4 of A_2A_R, MNYMVYFNFFACVLVPLLLMLGVYLYGRKKRRQRRR for TM5 of A_2A_R, YGRKKRRQRRRLAIIVGLFALCWLPLHIINCFTFF for TM6 of A_2A_R, LWLMYLAIVLSHTNSVVNPFIYAYYGRKKRRQRRR for TM7 of A_2A_R.
- YGRKKRRQRRRILGIWAVSLAIMVPQAAVME for TM4 of OX_1_R, SSFFIVTYLAPLGLMAMAYFQIFYGRKKRRQRRR for TM5 of OX_1_R, YASFTFSHWLVYANSAANPIIYNFYGRKKRRQRRR for TM7 of OX_1_R

### Bimolecular fluorescence complementation assay (BiFC)

HEK-293 T cells were transiently transfected with equal amounts of the cDNA for fusion proteins of the hemi-truncated Venus (1 μg of each cDNA). At 48 h after transfection, cells were treated for 4 h at 37° with medium or TAT peptides (4 μM) before plating 20 μg of protein in 96-well black microplates (Porvair, King’s lynn, UK). To quantify reconstituted YFP Venus expression, fluorescence at 530 nm was read in a Fluoro Star Optima Fluorimeter (BMG Labtechnologies, Offenburg, Germany) equipped with a high-energy xenon flash lamp, using a 10 nm bandwidth excitation filter at 400 nm reading. Protein fluorescence expression was determined as fluorescence of the sample minus the fluorescence of cells not expressing the fusion proteins (basal). Cells expressing receptor-cVenus and nVenus or receptor-nVenus and cVenus showed similar fluorescence levels than untransfected cells.

### Bioluminescence resonance energy transfer (BRET)

HEK-293 T cells were transiently transfected with a constant amount of cDNA for Rluc fusion proteins and increasing amounts of cDNA for YFP fusion proteins. At 48 h after transfection, 20 μg of cell suspension were plated in 96-well black microplates for fluorescence detection or in 96-well white microplates for BRET readings and Rluc quantification. YFP fluorescence at 530 nm was quantified in a Fluoro Star Optima Fluorimeter as described above. BRET signal was collected 1 min after addition of 5 μM coelenterazine H (Molecular Probes, Eugene, OR, USA) using a Mithras LB 940. The integration of the signals detected in the short-wavelength filter at 485 nm and the long-wavelength filter at 530 nm was recorded. To quantify protein-RLuc expression, luminescence readings were also performed after 10 minutes of adding 5 μM coelenterazine H. The net BRET is defined as (long-wavelength emission/short-wavelength emission)–Cf, where Cf corresponds to long-wavelength emission/short-wavelength emission for the donor construct expressed alone in the same experiment. BRET is expressed as milli-BRET units (net BRET × 1000). To calculate maximum BRET (BRET_max_) from saturation curves, data were fitted to a nonlinear regression equation, assuming a single-phase saturation curve with GraphPad Prism software (San Diego, CA, USA).

### Proximity ligation assay (PLA)

HEK293T cells were grown on glass coverslips and fixed in 4% paraformaldehyde for 15 min, washed with phosphate-buffered saline containing 20 mM glycine, permeabilized with the same buffer containing 0.05% Triton X-100, and successively washed with tris-buffered saline. Heteromers were detected using the Duolink II in situ PLA detection Kit (OLink; Bioscience, Uppsala, Sweden) following supplier’s instructions. A mixture of the primary antibodies (mouse anti-A_2A_R antibody (1:100; 05-717, Millipore, Darmstadt, Germany; RRID:AB_309931) and rabbit anti-A_1_R antibody (1:100; ab82477, Abcam, Bristol, UK; RRID: AB_2049141)) was used to detect A_1_-A_2A_Het together with PLA probes detecting mouse or rabbit antibodies. Then, samples were processed for ligation and amplification with a Detection Reagent Red and were mounted using a DAPI-containing mounting medium. Samples were analyzed in a Leica SP2 confocal microscope (Leica Microsystems, Mannheim, Germany) equipped with 405 nm and 561 nm laser lines. For each field of view, a stack of two channels (one per staining) and 4–6 Z-stacks with a step size of 1 μm were acquired. Images were opened and processed with Image J software (National Institutes of Health, Bethesda, MD, USA).

### cAMP determination assays

HEK-293 T cells expressing adenosine receptors were incubated for 4 h in serum-free medium containing 50 μM zardeverine. Cells were plated in 384-well white microplates (1500 cells/well), pre-treated with toxins or the corresponding vehicle for the indicated time, stimulated with agonists for 15 min before adding medium or 0.5 μM forskolin, and incubated for an additional 15 min. cAMP production was quantified by a TR-FRET (Time-Resolved Fluorescence Resonance Energy Transfer) methodology using the LANCE Ultra cAMP kit (PerkinElmer) and fluorescence at 665 nm was analyzed on a Pherastar Flagship Microplate Reader (BMG Labtech, Ortenberg, Germany).

### Dynamic mass redistribution (DMR) assays

The heteromer-induced cell signaling signature was determined using an EnSpire^®^ Multimode Plate Reader (PerkinElmer, Waltham, MA, USA) by a label-free technology. Refractive waveguide grating optical biosensors, integrated in 384-well microplates, allow extremely sensitive measurements of changes in local optical density in a detecting zone up to 150 nm above the surface of the sensor. Cellular mass movements induced upon receptor activation were detected by illuminating the underside of the biosensor with polychromatic light and measured as changes in wavelength of the reflected monochromatic light, which is a sensitive function of the index of refraction. The magnitude of this wavelength shift (in picometers) is directly proportional to the amount of DMR. Briefly, 24 h before the assay, cells were seeded at a density of 7500 cells per well in 384-well sensor microplates with 40 μL growth medium and cultured for 24 h (37 °C, 5% CO_2_) to obtain 70–80% confluent monolayers. Previous to the assay, cells were pre-treated with medium or toxins as indicated and incubated for 2 h in 40 μL per well of assay-buffer (HBSS with 20 mM HEPES, pH 7.15) in the reader at 24 °C. Thereafter, the sensor plate was scanned and a baseline optical signature was recorded prior to addition of 10 μL of receptor agonist dissolved in assay buffer containing 0.1% DMSO. DMR responses were monitored for at least 8000 s and data were analyzed using EnSpire Workstation Software v. 4.10.

### Computational modeling

The structural model of the A_1_-A_2A_Het bound to G_s_ (closed α_s_AH domain) and G_i_ (closed α_i_AH domain) was taken from our previous work [5]. This previous structural model contains a A_2A_R-based homology model of A_1_R. The structure of the adenosine A_1_R has recently been revealed [31], showing a remarkably similar structure (Additional file 1: Figure S6A). This structure of A_1_R contains a TM4/5 dimer interface that is in close agreement with our model (Additional file 1: Figure S6B). An intermediate conformation (obtained using the g_morph tool of the GROMACS package [32]) between the closed αAH domain (PDB id 1AZT) and the conformation observed in the crystal structure of the β_2_-AR in complex with Gs (PDB id 3SN6) was used to model the open αAH domain (Additional file 1: Figure S6C). This conformation is supported by DEER spectroscopy, deuterium-exchange and electron microscopy data [11–13]. The active state of β-arrestin-2 was built using a multi-template alignment combining the structure of the active β-arrestin-1 (PDB id 4JQI) [21] and the structure of rhodopsin in complex with visual β-arrestin (PDB id 4ZWJ) [14]. Structural models of the A_1_-A_2A_Het bound to β-arrestin-2 were modeled using the crystal structure of rhodopsin bound to β-arrestin (PDB id 4ZWJ) [14]. The structure of TM6 of A_2A_R fused to the cell-penetrating TAT peptide was modeled from the structure of A_2A_R. Molecular models of the A_1_-A_2A_Het with the TAT-fused TM6 peptide, disrupting the heteromer interface between A_1_R and A_2A_R, in complex with G_s_ (open α_s_AH domain) and G_i_ (open α_i_AH domain), was built from the structure of A_1_-A_2A_Het. The conformation of the proximal C-tail of A_2A_R (Ser305-Ala317) was modeled based on squid rhodopsin [33]. The remaining part of the C-tail (1Gly319–Ser412), cannot be unambiguously determined and it was modeled as suggested for the oxoeicosanoid receptor (OXER) [20], together with the structure derived from the human V2 vasopressin receptor in complex with β-arrestin-2 [21] (see Additional file 1: Figure S4 for details). Additional file 2: Table S1 shows the template structures used in the protein models. Modeller 9.12 was used to build these models [34]. The molecular models of A_1_-A_2A_Het in complex with G_s_ and G_i_ or β-arrestin, in the absence or presence of the TAT-fused TM6 peptide, were embedded in a pre-equilibrated box containing a lipid bilayer (~800 POPC molecules) with explicit solvent (~110,000 waters) and 0.15 M concentration of Na^+^ and Cl^–^ (~1800 ions). These initial complexes were energy-minimized and subsequently subjected to a 21 ns MD equilibration, with positional restraints on protein coordinates. These restraints were released and 500 ns of MD trajectory were produced at constant pressure and temperature. Computer simulations were performed with the GROMACS 4.6.3 simulation package [32], using the AMBER99SB force field as implemented in GROMACS and Berger parameters for POPC lipids. This procedure has been previously validated [35].

## Additional files

Additional file 1: **Figures S1–S6. Figure S1.** Control experiments on the effect of interfering peptides on the A_1_-A_2A_Het structure and G_s_ and G_i_ coupling to A_1_-A_2A_Het. **Figure S2.** Receptor signaling through A_1_R and A_2A_R. **Figure S3.** Recruitment of β-arrestin-2 by the A_1_-A_2A_Het. **Figure S4.** Modeling the orientation of the C-tail of A_2A_R. **Figure S5.** The influence of the C-terminal domain of A_2A_R in the signaling properties of the A_1_-A_2A_Het in the presence of pertussis toxin. **Figure S6.** Modeling A_1_R homodimer and α_s_AH and α_i_AH in closed and open conformations. (DOCX 5727 kb)

Additional file 2: **Table S1.** List of target sequences and template structures used to construct the computer models of A_1_-A_2A_Het in complex with G_i_ and G_s_. (PDF 23 kb)
