# Supplementary material for: Cross-communication between Gi and Gs in a G-protein-coupled receptor heterotetramer guided by a receptor C-terminal domain
Source: BMC Biol. 2018 Feb 28;16:24. doi: 10.1186/s12915-018-0491-x (PMC6389107; doi:10.1186/s12915-018-0491-x)
Supplement: Supplementary file 1 — Figures S1–S6. Figure S1. Control experiments on the effect of interfering peptides on the A1-A2AHet structure and Gs and Gi coupling to A1-A2AHet. Figure S2. Receptor signaling through A1R and A2AR. Figure S3. Recruitment of β-arrestin-2 by the A1-A2AHet. Figure S4. Modeling the orientation of the C-tail of A2AR. Figure S5. The influence of the C-terminal domain of A2AR in the signaling properties of the A1-A2AHet in the presence of pertussis toxin. Figure S6. Modeling A1R homodimer and αsAH and αiAH in closed and open conformations. (DOCX 5727 kb) [file 12915_2018_491_MOESM1_ESM.docx]

**Additional file 1**

Cross-communication between G_i_ and G_s_ in a G-protein-coupled receptor heterotetramer guided by a receptor C-terminal domain

Gemma Navarro^1,2,3^*, Arnau Cordomí^4^*, Marc Brugarolas^1,2,3^, Estefanía Moreno^1,2,3^, David Aguinaga^1,2,3^, Laura Pérez-Benito^4^, Sergi Ferre^5^, Antoni Cortés^1,2,3^, Vicent Casadó^1,2,3^, Josefa Mallol^1,2,3^, Enric I. Canela^1,2,3^, Carme Lluís^1,2,3^, Leonardo Pardo^4,&^, Peter J. McCormick^1,2,3,6,&^ and Rafael Franco^3,&^

*^1^Centro de Investigación Biomédica en Red sobre Enfermedades Neurodegenerativas. ^2^Institute of Biomedicine of the University of Barcelona (IBUB). ^3^Department of Biochemistry and Molecular Biology, Faculty of Biology, University of Barcelona, Barcelona, 08028 Spain; ^4^Laboratori de Medicina Computacional, Unitat de Bioestadística, Facultat de Medicina, Universitat Autònoma de Barcelona, 08193 Bellaterra, Spain; ^5^Integrative Neurobiology Section, National Institute on Drug Abuse, National Institutes of Health, Baltimore, MD 21224, USA; ^6^School of Veterinary Medicine, University of Surrey, Guildford GU2 7AL.*

*These authors contributed equally to this work

^&^These authors equally supervised this work

Corresponding authors:

Leonardo Pardo: leonardo.pardo@uab.es, Peter J. McCormick: p.mccormick@surrey.ac.uk, Rafael Franco: rfranco@ub.edu

Short Title: Cross-communication between G proteins

**
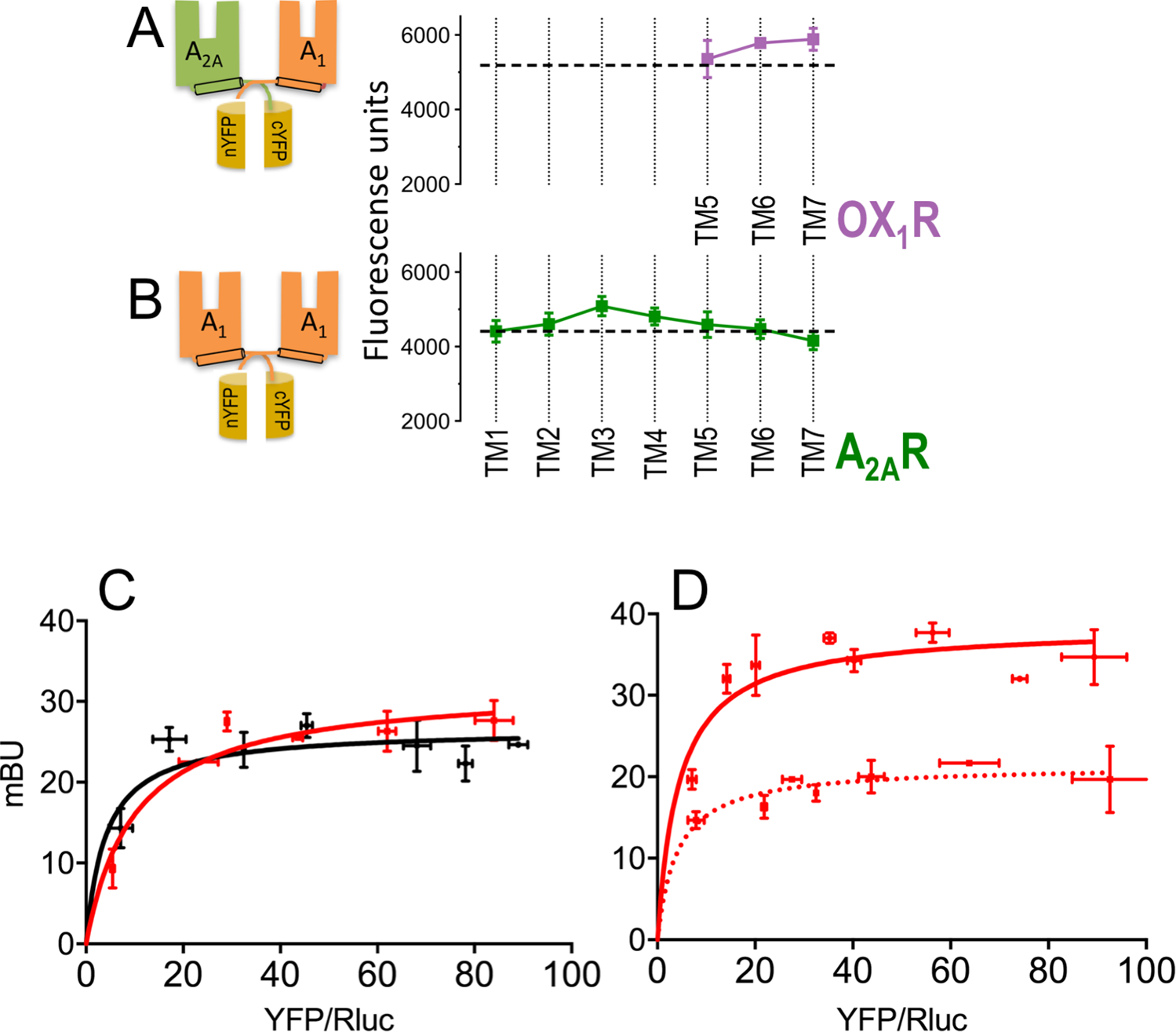
**

**Figure S1. Control experiments on the effect of interfering peptides on the A_1_-A_2A_Het structure and G_s_ and G_i_ coupling to A_1_-A_2A_Het**

In (A-B) BiFC assays were performed in HEK-293T cells transfected with cDNAs (1 µg) for A_1_R-nYFP and A_2A_R-cYFP (A) or A_1_R-nYFP and A_1_R-cYFP (B). Cells were pre-treated for 4 h with medium (control, broken lines) or with 4 µM of OX_1_R TM5 to TM7 synthetic peptides (A, purple squares) or A_2A_R TM1 to TM7 synthetic peptides (B, green squares). In each panel, there is a schematic representation of the BiFC pairs and conditions. In (C-D) BRET assays were carried out in HEK-293T cells expressing a constant amount of G_s_RLuc (red line in C and D) or G_i_RLuc (black line in C) and increasing amounts (0.1 to 0.7 µg cDNA) of A_2A_R-cYFP and A_1_R-nYFP. Cells were not treated (C) or treated (D) overnight with medium (solid line) or PTX (10 ng/mL, dotted line) and non-stimulated (C) or stimulated (D) with 100 nM CGS-21680 for 10 min. BRET saturation curves were obtained in which the relative amount of BRET is given as a function of 100 x the ratio between the fluorescence of the acceptor (YFP) and the luciferase activity of the donor (Rluc). BRET is expressed as milli BRET units (mBU) and are the mean ± SEM of 7 different experiments grouped as a function of the amount of BRET acceptor.

**
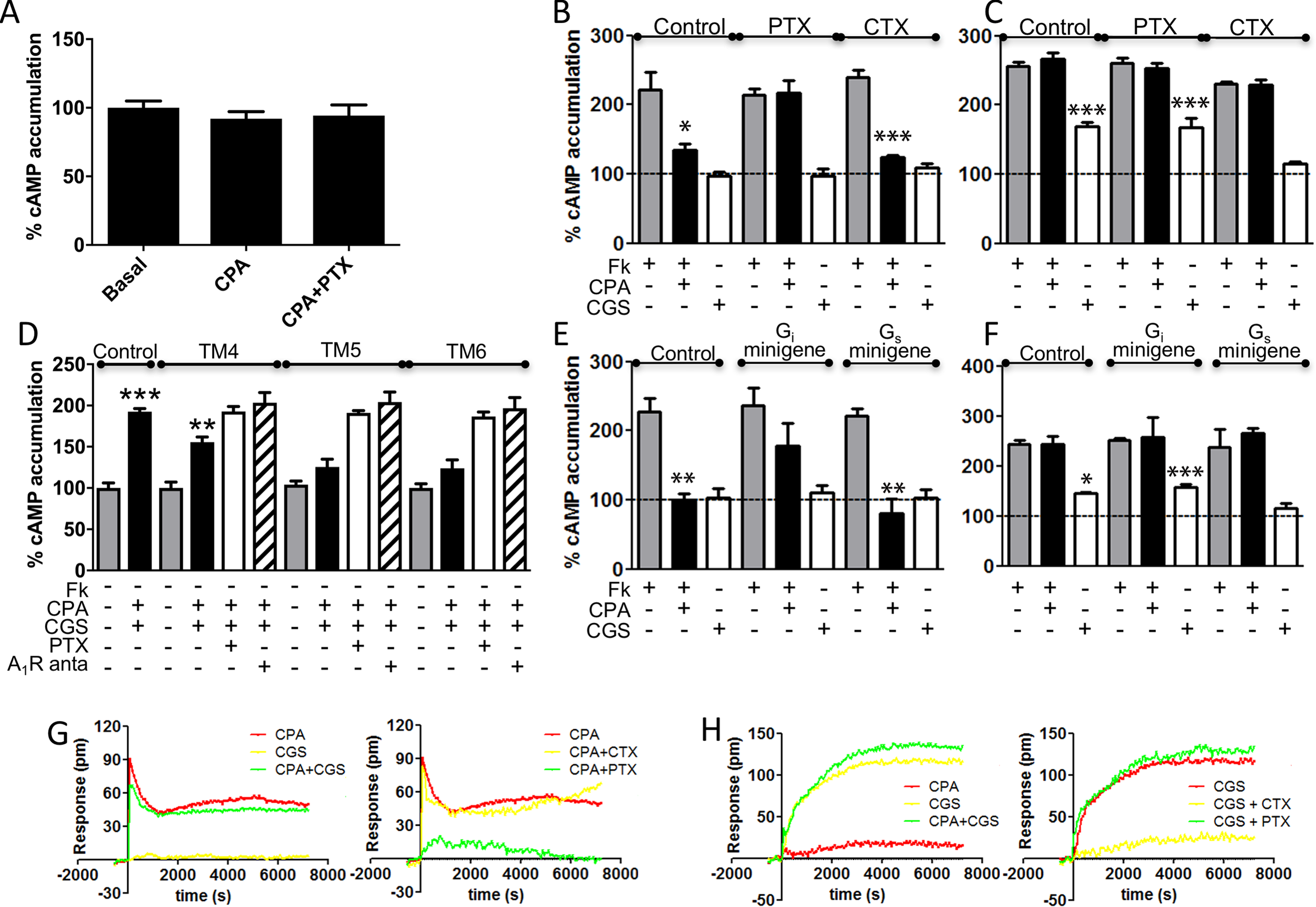
**

**Figure S2. Receptor signaling through A_1_R and A_2A_R**

In (A) cAMP determinations were performed in HEK-293T cells transfected with the cDNA (0.5 µg) for A_1_R and pre-treated overnight with medium or pertussis toxin (PTX, 10 ng/ml) and stimulated with 100 nM CPA in the absence of forskolin. In (B, C) cAMP determination were performed in HEK-293T cells transfected with the cDNA (0.5 µg) for A_1_R (B) or A_2A_R (C). Cells were pre-treated with medium (control), overnight with pertussis toxin (PTX, 10 ng/ml), or 1 h with cholera toxin (CTX, 100 ng/mL) and were no-stimulated (basal, dotted line) or stimulated with forskolin (Fk, 0.5 µM, gray bars), with forskolin and 100 nM of CPA (black bars) or with 100 nM of CGS-21680 (white bars). In (D) cAMP determinations were performed in HEK-293T cells transfected with the cDNAs for A_1_R (0.5 µg) and A_2A_R (0.5 µg) and pre-treated overnight with medium or pertussis toxin (PTX, 10 ng/ml, white bars) and then for 4h with vehicle (control) or with 0.4 µM of A_2A_R peptides TM4, TM5, TM6. Cells were treated, for 15 min with the A_1_R antagonist (scratched bars) or medium (black bars) and stimulated with CPA 100 nM and CGS-21680 (CGS, 100 nM) in the absence of forskolin. Values are the mean ± SEM of 7 experiments per group. One-way ANOVA followed by the Bonferroni’s *post hoc* test showed a significant effect of CPA plus CGS-21680 respect to basal (non-stimulated cells) (^*^p < 0.05, ^**^p < 0.01, ^***^p < 0.001). In (E, F) cells were transfected as in B, C respectively and not co-transfected (control) or co-transfected with the cDNAs (0.5 µg) for mini-genes coding for peptides that block the coupling to either G_s_ or G_i_. Cells were stimulated as indicated above. Increases in cAMP levels were expressed over basal. Values are the mean ± SEM of 6 experiments per group. One-way ANOVA followed by the Bonferroni’s *post hoc* test showed a significant effect respect to basal in samples only stimulated with CGS 21680 or respect to forskolin only stimulated cells in samples in the presence of forskolin (^*^p < 0.05, ^**^p < 0.01, ^***^p < 0.001). In (G, H) dynamic mass redistribution analyses were performed in cells transfected and stimulated as indicated in B and C. The resulting picometer-shifts of reflected light wavelength (pm) against time were monitored. Each curve is the means of a representative optical trace experiment carried out in triplicates.

**
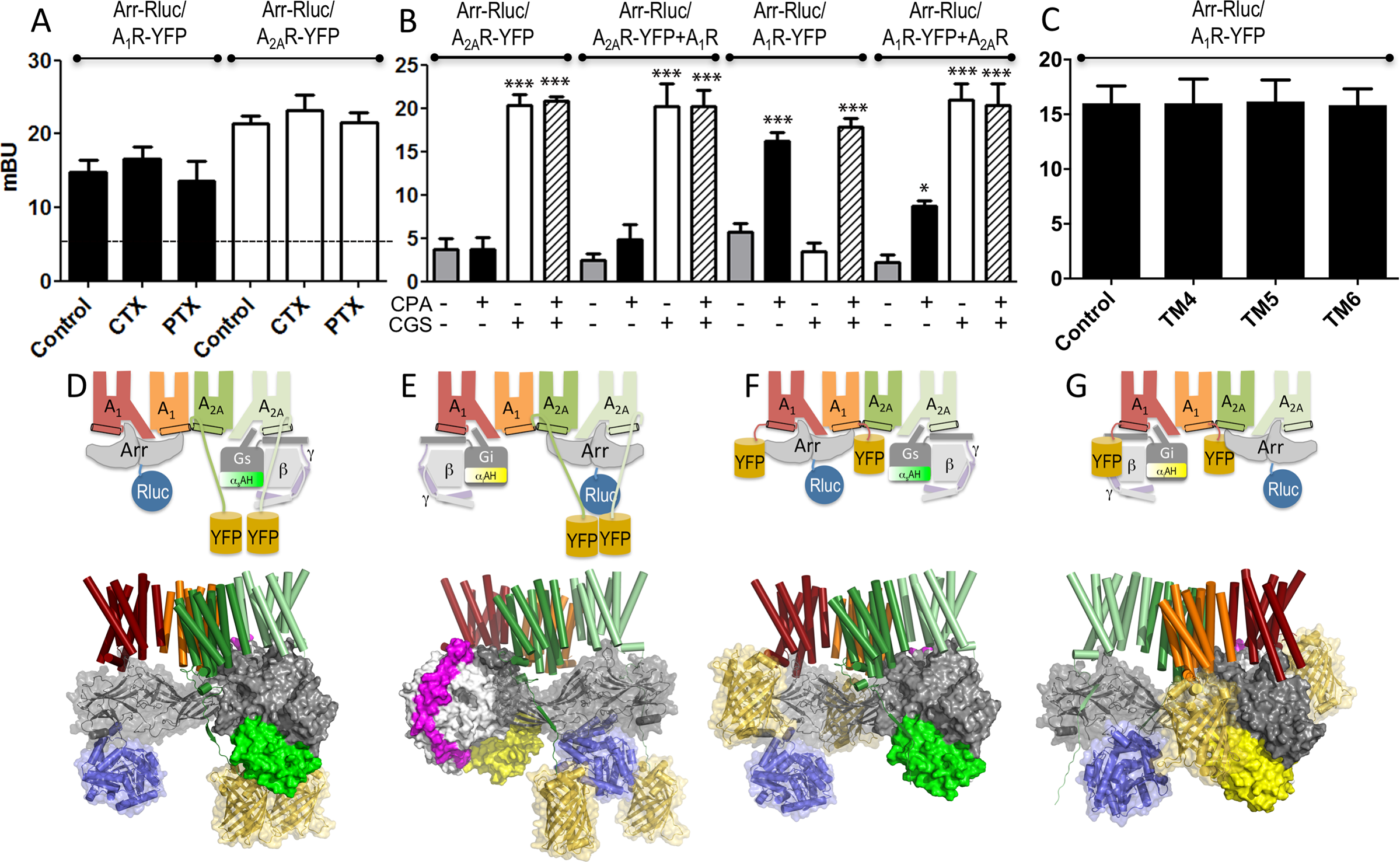
**

**Figure S3. Recruitment of β-arrestin-2 by the A_1_-A_2A_Het**

In (A, B) receptor agonist-induced β-arrestin-2 recruitment was measured by BRET experiments. HEK-293T cells were transfected with the cDNAs for β-arrestin-2-Rluc (Arr-Rluc, 0.5 µg cDNA) and A_1_-YFP (0.4 µg cDNA), A_2A_-YFP (0.4 µg cDNA), A_1_R (0.4 µg cDNA) or A_2A_R (0.4 µg cDNA) as indicated in the panels. Cells were not treated (control in A or B) or treated overnight with pertussis toxin (PTX, 10 ng/ml) or cholera toxin (CTX, 100 ng/mL). Cells were stimulated with medium (basal, dotted line in panel A and gray bars in panel B), the A_1_R agonist CPA (100nM, black bars), the A_2A_R agonist CGS-21680 (CGS, 100nM, white bars) or both (scratched bars). Positive BRET was expressed as mBU (see Methods). Values are the mean ± SEM of 7 experiments per condition. One-way ANOVA followed by the Bonferroni’s *post hoc* test showed significant differences over basal (***p<0.001). In (B) positive BRET was observed when cells expressing Arr-Rluc and A_1_R-YFP were activated with the A_1_R agonist CPA but not with the A_2A_R agonist CGS-21680 and CGS-21680 did not modify the CPA signaling. Analogously, positive BRET was observed when cells expressing Arr-Rluc and A_2A_R-YFP were activated with CGS-21680 but not with CPA and CPA did not modify CGS-21680 signaling. However, in cells expressing Arr-Rluc, A_1_R-YFP and non-fused A_2A_R, the ability of CPA to increase BRET by recruitment of β-arrestin-2 to A_1_R, was diminished compared to cells not expressing A_2A_R. These results suggest that the presence of A_2A_R-G_s_ in the heteromer modifies the position of YFP fused to the C-terminal domain of A_1_R or/and Rluc fused to the C-term of β-arrestin-2 in a way that energy transfer diminished. Moreover, CGS-21860 was able to increase BRET in cells expressing Arr-Rluc, A_1_R-YFP and non-fused A_2A_R to a similar level to cells only expressing Arr-Rluc and A_2A_R-YFP (white bars). This indicates that Arr-Rluc bound to non-fused A_2A_R can energy transfer to the arrestin-unbound A_1_R-YFP acceptor. Energy transfer is not observed when CPA binds to non-fused A_1_R in cells expressing Arr-Rluc, A_2A_R-YFP and non-fused A_1_R (black bars). In (C) HEK-293T cells were transfected with the cDNAs for β-arrestin-2-Rluc (Arr-Rluc, 0.5 µg cDNA) and A_1_R-YFP (0.4 µg cDNA) and pre-treated for 4h with vehicle (control) or with 0.4 µM of A_2A_R transmembrane peptides TM4, TM5, TM6 and stimulated with CPA 100 nM. Values are the mean ± SEM of 6 experiments per condition. One-way ANOVA followed by the Bonferroni’s *post hoc* test showed no significant differences over control. In (D-G) molecular models of Arr-Rluc, A_2A_R-YFP and non-fused A_1_R (C, D) or Arr-Rluc, A_1_R-YFP and non-fused A_2A_R (E, F) with Arr-Rluc bound to A_1_R (C, E) or A_2A_R (D, F) and G_i_ bound to A_1_R (D, F) or G_s_ bound to A_2A_R (C, E) were given to explain the above described results. These molecular models show that Arr-Rluc bound to A_1_R cannot transfer energy to A_2A_R-YFP because the long C-tail of A_2A_R position YFP near the α_s_AH domain or the N-term of the γ−subunit (see Fig. S4), far from Rluc (panel C). In contrast, Arr-Rluc bound to A_2A_R can transfer energy to A_1_R-YFP because the short C-tail of the α_i_-unbound A_1_R protomer positions YFP in close proximity to Rluc (panel F, which is rotated 180º relative to panels C-E to visualize the proximity of YFP and Rluc).

**
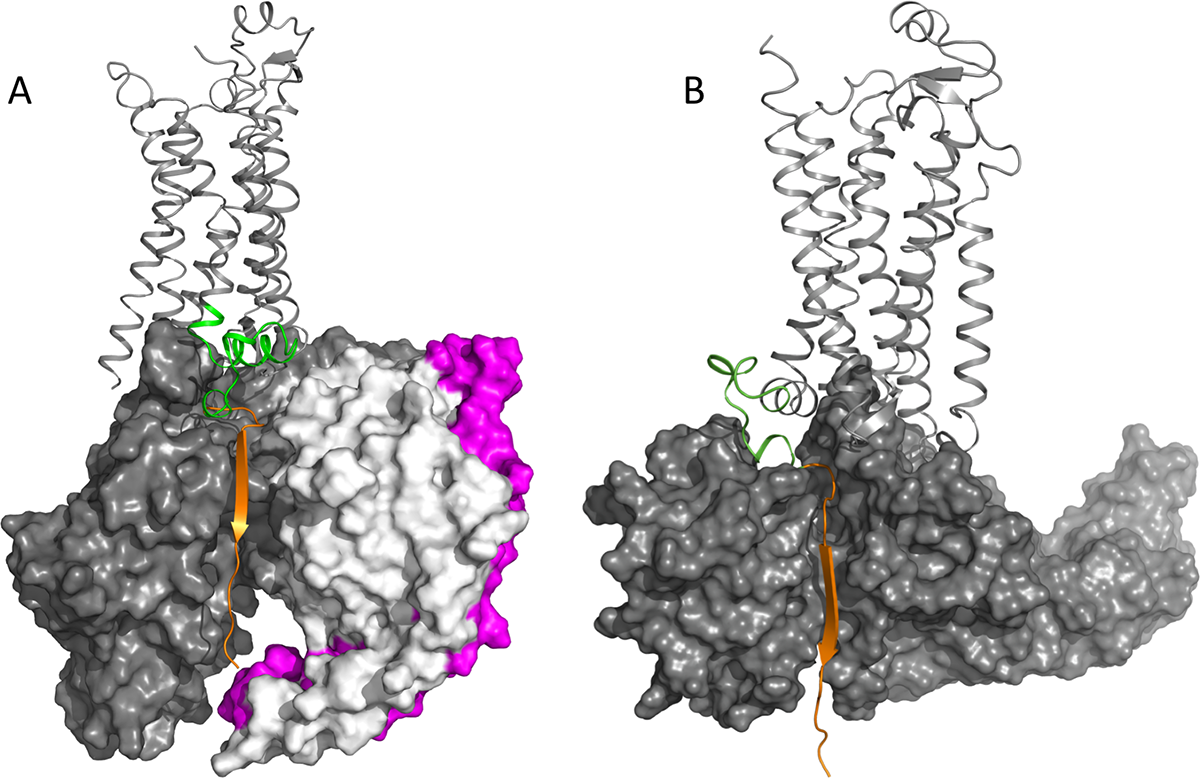
**

**Figure S4. Modeling the orientation of the C-tail of A_2A_R**

Although the exact conformation of the A_2A_R C-tail (102 amino acids, Gln311-Ser412) cannot unambiguously be determined, its orientation was modeled using a combination of structural templates. First, the C-tail of squid rhodopsin [35], which contains the conserved amphipathic helix 8 that runs parallel to the membrane and an additional cytoplasmic helix 9, was used to model 13 amino acids (Ser305-Ala317 in green). Second, the C-tail of the human V2 vasopressin receptor, determined in complex with β-arrestin-2 [21], was used to model 18 amino acids (Gly318-Ser335 in orange). The laboratory of Kostenis has shown that the C-term of OXER, labeled with Rluc (OXER-Rluc), gets close to the N-term of the γ-subunit, labeled with GFP (γ-GFP) [20]. Thus, the modeled orientation of the C-tail of A_2A_R accomplishes simultaneously two restraints imposed by the G protein and arrestin: it is positioned near the N-termini of the γ-subunit of G_s_ (in purple) as suggested for the OXER (panel A) and it extends the N-domain β-sandwich fold of arrestin, binding as an antiparallel β-strand, as suggested for V2 vasopressin receptor (panel B).


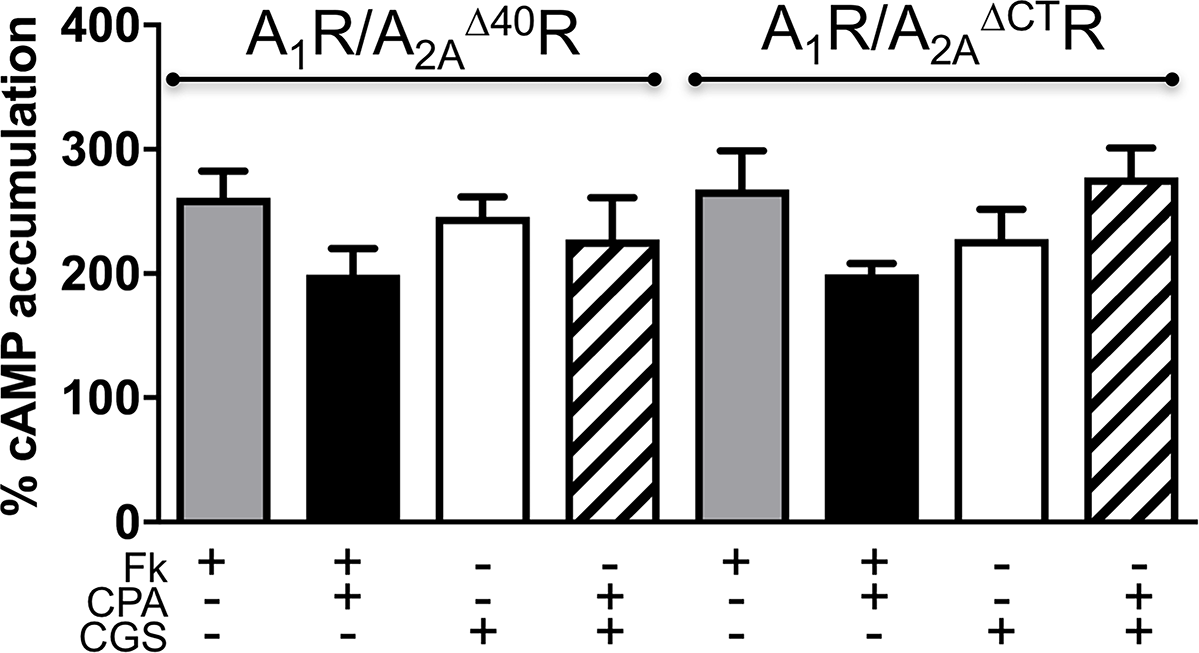


**Figure S5. The influence of the C-terminal domain of A_2A_R in the signaling properties of the A_1_-A_2A_Het in the presence of pertussis toxin**

HEK-293T cells expressing A_2A_^Δ40^R (0.3 µg cDNA transfected) or A_2A_^ΔCT^R (0.3 µg cDNA transfected) and A_1_R (0.4 µg cDNA transfected) were pre-treated overnight with pertussis toxin (PTX, 10ng/ml) before stimulation with forskolin (Fk, 0.5 µM, gray bars), with forskolin and the A_1_R agonist CPA (100 nM, black bars), with the A_2A_R agonist CGS-21680 (CGS, 100 nM, white bars) or with CPA and CGS 21680 (striped bars). Increases in cAMP levels were expressed over those in unstimulated cells (100% basal). Values are the mean ± SEM of 7 experiments per group. The results confirm that PTX blocks the ability of CPA to decrease both the forskolin-induced and the CGS-induced cAMP accumulation.

**
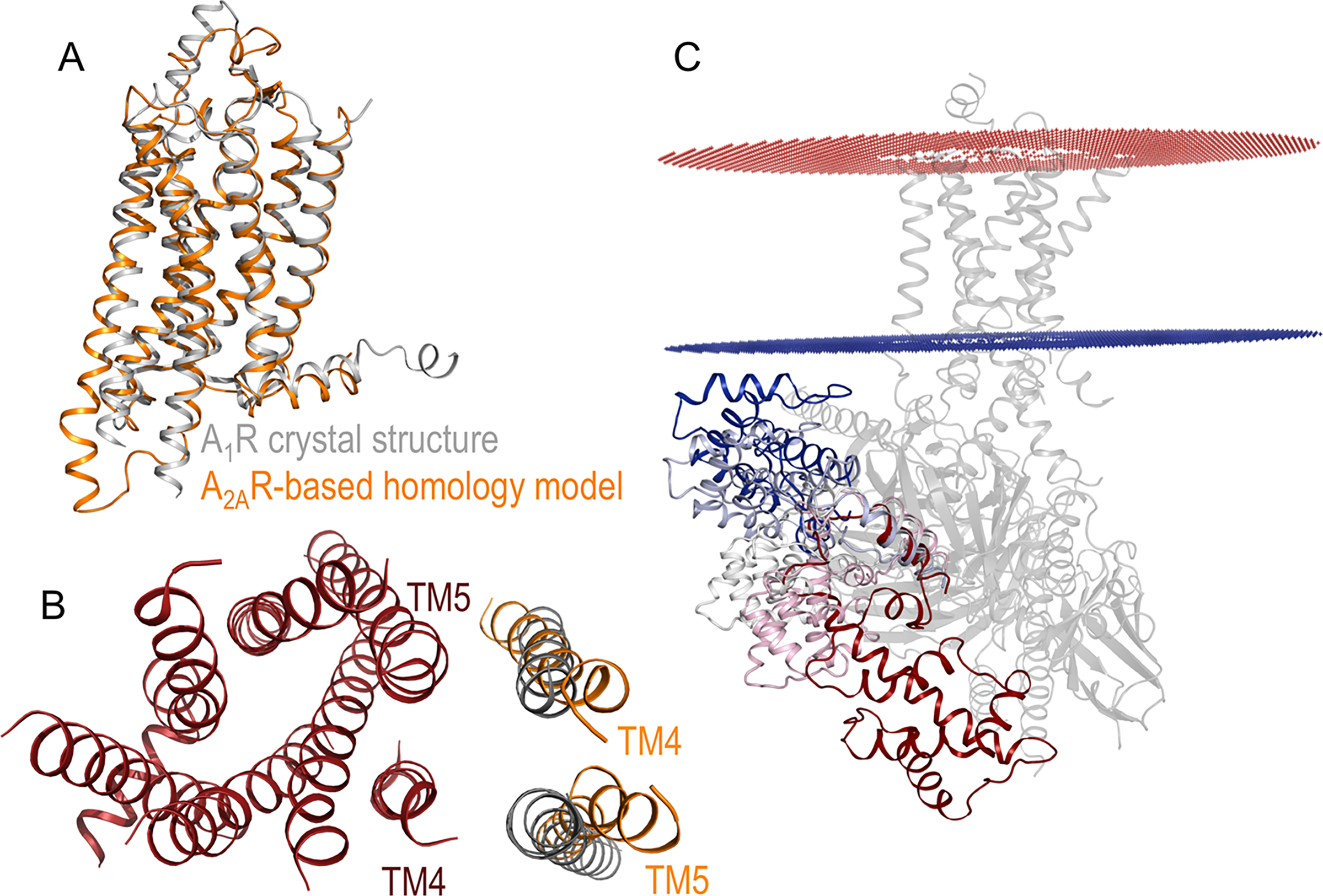
**

**Figure S6. Modeling A_1_R homodimer and α_s_AH and α_i_AH in closed and open conformations**

In (A) comparison of the recently published crystal structure of A_1_R (PDB id 5UEN) [31] our A_2A_R-based homology model of A_1_R [5]. In (B) superimposition of the TM4/5 interface obtained in the crystal structure of A_1_R (red and gray) [31] and in the model (orange) [5]. In (C) the α_s_AH domain of the α_s_-subunit in the crystal structure of the β_2_-AR in complex with G_s_ (in blue) (PDB id 3SN6) and in a homology model of the α_s_-subunit in the closed conformation (PDB id 1AZT) (red) are shown. Intermediate conformations (light blue, white, light red) between these two structures were obtained using the g_morph tool of the GROMACS package. The open conformation of the α_s_AH domain (white) was selected from these intermediate conformations in such a manner that the distance (d[Asn112α_s_-Asn261α_s_]) between the Cα atoms of Asn112 (in the α_s_AH domain) and Asn261 (Ras domain) is ~40Å, as observed in DEER distance distributions between spin labels attached to these amino acids [13]. Similar procedure was used to model open and closed conformations of the α_i_-subunit (not shown). The selected open conformation of α_i_AH also reproduces a distance (d[Arg90α_i_-Glu238α_i_]) between the Cα atoms of Arg90 (α_i_AH domain) and Glu238 (Ras domain) of ~40Å [13].
