# Supplementary material for: Cross-communication between Gi and Gs in a G-protein-coupled receptor heterotetramer guided by a receptor C-terminal domain
Source: BMC Biol. 2018 Feb 28;16:24. doi: 10.1186/s12915-018-0491-x (PMC6389107; doi:10.1186/s12915-018-0491-x)
Supplement: Supplementary file 2 — Table S1. List of target sequences and template structures used to construct the computer models of A1-A2AHet in complex with Gi and Gs. (PDF 23 kb) [file 12915_2018_491_MOESM2_ESM.pdf]

Table S1. List of target sequences and template structures used to construct the computer models of A_1-A_{2A}Het in complex with G_i and G_s.

Protein model	UniProt ID	PDB IDs
A_{2A}R (G_s-unbound)	P29274	**4EIY**, except ICL3 (209-218: 2Z73)
A_{2A}R (G_s-bound)	P29274	**4EIY**, except TMs 5-6 (179-259: 3SN6), ICL3 (209-218: 2Z73) and C-term (305-317: 2Z73, 318-335: 4JQI)
A_1R (G_i-unbound)	P30542	(based on A_{2A}R G_s-unbound)
A_1R (G_i-bound)	P30542	(based on A_{2A}R G_s-bound)
$G_{\alpha s}$	P04896	**3SN6**, except AH (56-208: **1AZT**)
$G_{\alpha i}$	P10824	**3SN6**, except AH (49:186: **1AGR**)
G_{β}	P54311	**3SN6** (1-340)
$G\gamma$	P63212	**3SN6** (5-62)
β-arrestin-1	P29066	N-term (7-174: **4ZWJ**), C-term (175-360: **4JQI**)
Rluc	P27652	**2PSD** (4-308)
YFP	Q963I9	**2RH7** (7-226)
